# The PROTECTOR strategy employs dCas orthologs to sterically shield off-target sites from CRISPR/Cas activity

**DOI:** 10.1038/s41598-023-29332-2

**Published:** 2023-02-09

**Authors:** Daniel M. Sapozhnikov, Moshe Szyf

**Affiliations:** Department of Pharmacology and Therapeutics, Faculty of Medicine and Health Sciences, McGill University, Montreal, QC Canada

**Keywords:** Genetic engineering, Genetic techniques

## Abstract

Off-target mutagenesis of CRISPR/Cas systems must be solved to facilitate safe gene therapy. Here, we report a novel approach, termed "PROTECTOR", to shield known off-target sites by directing the binding of an orthologous nuclease-dead Cas protein to the off-target site to sterically interfere with Cas activity. We show that this method reduces off-target mutation rates of two well-studied guide RNAs without compromising on-target activity and that it can be used in combination with high-fidelity Cas enzymes to further reduce off-target editing. This expands the suite of off-target mitigation strategies and offers an ability to protect off-target sites even when their sequences are fully identical to target sites.

## Introduction

Only a decade since its discovery as a gene-editing technology, CRISPR/Cas is being broadly tested in human trials. So far, however, the greatest concern about its long-term safety has yet to be resolved: CRISPR/Cas tolerates sequence mismatches and edits off-target sites at a high frequency^1,2^, thereby posing significant clinical risk of producing adverse effects by unintended mutagenesis. There are now several strategies to reduce off-target effects of CRISPR/Cas (reviewed in Naeem et al.^3^), most notably the development of rationally engineered high-fidelity Cas variants^4–8^ and “dead” truncated gRNAs that inhibit off-target cleavage^9,10^. These techniques can be highly effective, but they do not cover all off-target scenarios. “Dead” gRNAs fail to prevent off-target editing at many sites and are mechanistically unsuitable for blocking gRNA-driven off-targets of dCas-based epigenetic editors and base editors^9,10^. Similarly, all high-fidelity Cas enzymes–while effective at reducing off-target effects with most gRNAs–can completely fail to reduce off-target mutagenesis with other gRNAs and can all cleave sequences with up to five mismatches^11–14^. Furthermore, the majority of engineered Cas9 enzymes exhibit reduced on-target activity compared to wild-type *S. pyogenes* Cas9 (SpCas9)^4–6,8,12,15^ and variants with comparable activity to SpCas9^6,12^ display milder reductions of off-target activity. While these global multi-target comparisons describe general trends, the specific efficiencies at both on- and off-target sites of all engineered variants are gRNA/target-dependent and these variants often fail to cleave specific targets that can be cleaved by wild-type SpCas9 while also failing to reduce off-target mutagenesis at specific sites with specific gRNAs^4,12^. Therefore, there remains a need to develop new methods to reduce CRISPR/Cas off-target effects.

Here, we present a complementary new strategy for the mitigation of off-target effects which has unique benefits and can be combined with existing strategies to further improve CRISPR/Cas efficacy and specificity. At both target and off-target sites, active Cas nuclease complexed with a specific gRNA requires access to the DNA to first identify protospacer adjacent motifs (PAMs) and then interrogate the neighboring sequence for complementarity to the gRNA sequence prior to cleavage^16^. This suggests that a previously identified off-target site for any given gRNA can be obscured from active Cas and gRNA by specifically restricting the physical accessibility of the known off-target region, such that active Cas is no longer able to interrogate the sequence at the off-target site, resulting in reduced off-target activity. One option to restrict access to specific DNA sequences is through the use of a nuclease-dead Cas variant (dCas)–which binds to but does not cleave target sequences–and has been previously used to sterically interfere with RNA polymerase^17^ and DNA methyltransferases^18^ at specific targets. We hypothesized that dCas could similarly be targeted to a known off-target site in order to outcompete and interfere with catalytically active Cas and therefore specifically reduce off-target activity. Application of such a strategy requires a priori knowledge of off-target sites of a known gRNA and it is therefore most suitable for gRNAs with extensively studied off-target profiles by low-bias methods (e.g. GUIDE-seq^19^), such as gRNAs intended for clinical use.

**Figure 1:**
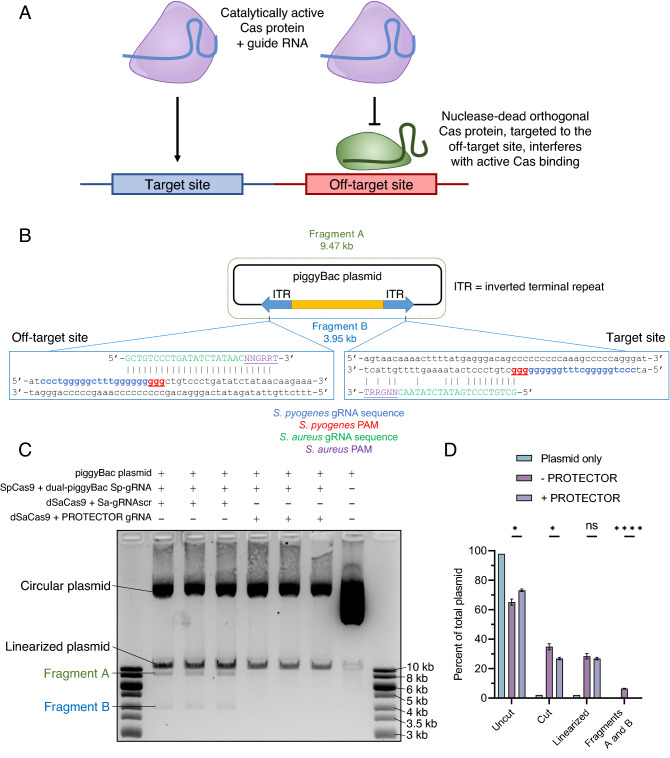
PROTECTOR schematic and proof-of-principle. (**A**) A schematic representation of the PROTECTOR strategy, showing that specific targeting of a dCas ortholog to a known off-target site will prevent active Cas from accessing the site, while not affecting the ability of Cas to cleave target DNA. (**B**) Representation of plasmid DNA used as substrate for the in vitro PROTECTOR assay in (**C**). The plasmid has two sequences that are targeted by an *S. pyogenes* gRNA (in blue with PAM in red and underlined) within two inverted terminal repeats (ITRs). The *S. aureus* gRNA sequence designed to be specific to only the off-target site is represented in green (with PAM in purple and underline). It targets only the site marked as “off-target” and has extensive mismatching with the target site such that it should not direct binding of *S. aureus* dCas9 to the target and only obstruct *S. pyogenes* Cas9 at the off-target site. Cleavage of the plasmid by SpCas9/Sp-gRNA at both the target and off-target sites results in the generation of two products, referred to as Fragment A and Fragment B, whereas cleavage by SpCas9/Sp-gRNA at only the target site results in linearization of the plasmid. (**C**) Agarose gel electrophoresis results of in vitro PROTECTOR assay. ~ 1.5 µg piggyBac plasmid DNA (from (**B**)) was incubated with either a control reaction (recombinant dSaCas9 and an in vitro transcribed scrambled non-targeting Sa-gRNA: Sa-gRNAscr) or a PROTECTOR reaction (recombinant dSaCas9 and in vitro transcribed PROTECTOR-gRNA depicted in (**B**)) prior to the addition of catalytically active SpCas9 protein and the Sp-gRNA targeting the two sites in the plasmid as depicted in (**B**) (see Methods). The results show that the SpCas9/Sp-gRNA ribonucleoprotein complex cleaves at both target sites in the control reaction, as seen by the presence of bands representing Fragments A and B, but addition of the PROTECTOR components prevents cleavage at only the off-target site, as visualized by the generation of only linearized plasmid from the circularized template and absence of Fragments A and B. The marker in the first and last lane is GeneRuler 1 kb DNA ladder (Thermo Fisher) loaded at a volume of 10 µL and 7 µL in the first and last lanes, respectively. The results of (**C**) are quantified in (**D**) using ImageJ. Here, “uncut” refers to the circular form of the plasmid, indicating no cleavage by SpCas9 and "cut” refers to the sum total of all forms of the plasmid that have one or two cuts (linearized + Fragment A + Fragment B). Data are presented as mean ± SEM: “percent of total plasmid” is calculated as the intensity of the indicated band type divided by the sum of the intensities of all bands in the respective lane. Conditions with and without PROTECTOR components were compared by multiple independent t-tests with a correction for multiple comparisons by the FDR method (* indicates FDR < 0.05 and **** indicates FDR < 0.0001).

To prevent deleterious cross-talk–active Cas from using the gRNA intended for dCas and vice versa–it is critical for the two CRISPR/Cas systems to be completely orthogonal: that is, to only be capable of utilizing their own gRNAs and not those from the other system^20^. We therefore term this strategy “PROTECTOR”: PRevention of Off-Target Effects of CRISPR/Cas by Thwarting Orthologs (Fig. 1A). As presented throughout the manuscript, the term PROTECTOR refers to the strategy as a whole and involves two components–a dCas protein orthologous to the Cas9 protein used for gene editing and an ortholog-specific gRNA that guides this dCas protein–both of which can be referred to by their species-of-origin or by PROTECTOR dCas or PROTECTOR gRNA, respectively.

By strategically placing target/off-target mismatches at positions critical for Cas binding–particularly in the seed region and PAM^21^–the PROTECTOR strategy offers an ability to specifically bind and shield off-target sites without interfering with target site Cas activity. Though this strategy requires the presence of a PAM in proximity to the off-target site (specific to the dCas ortholog being used)–and, ideally, its absence from the target site–this is now highly feasible by careful ortholog selection in light of a diverse set of at least 900 natural homologs recognizing PAMs that cover 92% of all possible sequence variations over a 4 base-pair window^22,23^ as well as ongoing efforts to engineer PAM-less Cas variants which have so far yielded examples such as Cas9-NG and the near PAM-less SpRY variant^24,25^.

This strategy, in principle, offers two further novel capacities that are absent in recent “dead”/truncated gRNA off-target reduction strategies^9,10^: (1) specificity even when a given gRNA target sequence is completely identical to the off-target, by exploiting differences in flanking sequences and, (2) the blocking of off-targets of dCas-based epigenetic editors, which exhibit more gRNA-based off-target effects than active Cas because binding and cleavage activity requires more target sequence similarity than binding activity alone^21^.

In this work, we show that, in vitro, an orthologous dCas9 protein can interfere with the cleavage activity of an active Cas9 protein specifically at an artificial off-target site. We then demonstrate that the PROTECTOR strategy, when expressed stably in human cells, reduces off-target editing of two well-established gRNAs without interfering with on-target activity. We further show that the PROTECTOR strategy can be used alongside high-fidelity Cas9 variants to improve the gene editing outcomes in scenarios that are not fully resolved by the use of these variants. Finally, we report that inclusion of a KRAB domain in the PROTECTOR strategy does not improve its utility and that co-transfection rather than stable expression of PROTECTOR components still demonstrates success in reducing off-target effects.

## Results

### Orthologous dCas9 and gRNA interferes with CRISPR/Cas cleavage in vitro

To assess whether the dCas9 protein can physically interfere with cleavage by the Cas9 protein specifically at an off-target site, we first tested the PROTECTOR strategy in vitro using recombinant catalytically active Cas protein from *S. pyogenes* (SpCas9) and a fully orthogonal^20^ recombinant nuclease-dead Cas protein from *Staphylococcus aureus* (dSaCas9)^26^ as the PROTECTOR dCas component*.* We produced by in vitro transcription an *S. pyogenes* gRNA (Sp-gRNA) targeting a sequence that occurs twice in a piggyBac^27^-derived plasmid–as it bears two inverted terminal repeats (ITRs)–and thus resembles a worst-case gene-editing scenario wherein the off-target site is fully identical to the target site. However, the sequences that immediately flank the two sites are sufficiently dissimilar such that it is possible to design a PROTECTOR gRNA (*S. aureus* gRNA or Sa-gRNA) specifically directed to only the off-target site, where it overlaps the off-target Sp-gRNA region and protospacer adjacent motif (PAM) by 7 base-pairs and is thus predicted to sterically interfere with off-target SpCas9 binding and cleavage (Fig. 1B). At the target site, the PROTECTOR gRNA PAM site is completely absent and there are 8 additional mismatches throughout the gRNA sequence (Fig. 1B), so it cannot specifically interfere with on-target cleavage. Plasmid DNA was pre-incubated with either the PROTECTOR components or a non-targeting scrambled control Sa-gRNA (Sa-gRNAscr), followed by incubation with SpCas9 and the piggyBac-targeting Sp-gRNA. Here, pre-incubation with PROTECTOR components and an approximately 10:1 stoichiometric excess of PROTECTOR components relative to active SpCas9 components were used in order to ensure that PROTECTOR quantities were not limiting in this proof-of-mechanism experiment.

The control reaction produced two bands (fragment A (9.47 kb) & fragment B (3.95 kb)) that were the expected cleavage products that could only be generated by SpCas9/Sp-gRNA activity at both the on- and off-target sites (Fig. 1C,D). The off-target cut generating fragments A and B occurred at a frequency of at least 18% of all SpCas9-digested DNA (linearized plus fragments A and B). However, when the PROTECTOR gRNA was added to the reaction, the off-target site was no longer cleaved, as indicated by the generation of only linearized plasmid and complete absence of fragments A and B. Despite a comparable level of linearized product with and without PROTECTOR gRNA (Fig. 1D) there was a minor reduction in overall cleavage from 35 to 27% (~ 80% activity, *p* = 0.02113). This data serves as proof-of-principle that the PROTECTOR strategy successfully inhibits off-target CRISPR/Cas9 activity even when the off-target site is fully identical to the target site.

### The PROTECTOR strategy reduces off-target mutagenesis of a CCR5-targeting gRNA in human cells

**Figure 2:**
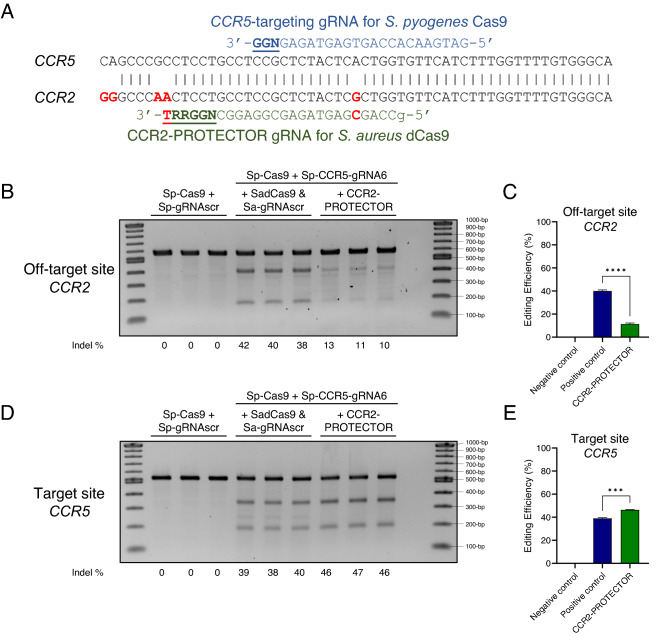
The PROTECTOR strategy applied to HEK293 cells transfected with CRISPR/Cas9 targeting *CCR5*. (**A**) Schematic showing the specificity of the PROTECTOR gRNA for the off-target locus based on the sequence comparison between the target of Sp-CCR5-gRNA6 in the *CCR5* gene and the off-target locus in the *CCR2* gene. Mismatches in the *CCR2* sequence are bolded and in red. Sp-CCR5-gRNA6 is depicted in blue with the PAM sequence bolded and underlined. The PROTECTOR gRNA is depicted in green with the PAM sequence bolded and underlined. Mismatches between the PROTECTOR gRNA sequence and the target site sequence are also bolded and in red. Agarose gel results of T7E1 assay for the off-target site in *CCR2* and target site in *CCR5* are depicted in (**B**) and (**D**). The first lane contains 1 kb Plus DNA Ladder (NEB), followed by results of T7E1 assays performed on the following biological triplicate sets of transfected samples: (1) summarized as “Negative control” in (**C**) and (**E**), HEK293 cells stably expressing dSaCas9 and PROTECTOR gRNA that were transfected with 250 ng SpCas9 plasmid and 250 ng scrambled non-targeting Sp-gRNA plasmid (Sp-gRNAscr), (2) summarized as “Positive control” in (**C**) and (**E**): HEK293 cells stably expressing dSaCas9 and scrambled non-targeting Sa-gRNA, transfected with 250 ng SpCas9 plasmid and 250 ng Sp-CCR5-gRNA6 plasmid, and (3) summarized as “CCR2-PROTECTOR” in (**C**) and (**E**), HEK293 cells stably expressing dSaCas9 and PROTECTOR gRNA, transfected with 250 ng SpCas9 plasmid and 250 ng Sp-CCR5-gRNA6 plasmid. The final two lanes contain a negative (water) control for the T7E1 assay followed by 1 kb Plus DNA Ladder. Indel estimates for each sample are depicted at the bottom of each lane, rounded to the nearest whole number. The results of (**B**) and (**D**) are quantified in (**C**) and (**E**), respectively. Data in (**C**) and (**E**) are presented as mean ± SEM and were quantified with ImageJ: editing efficiency is calculated as the intensity of T7E1 cleavage products divided by the sum of the intensities of T7E1 cleavage products and the uncut PCR amplicon. **** represents *p* < 0.0001 and *** represents *p* < 0.001 by independent t-test.

Next, we sought to determine if the PROTECTOR strategy could be applied to gene editing challenges in the human genome using previously published and therapeutically relevant gRNAs in cultured human cells. The *CCR5* gene is a clinical candidate for editing to confer HIV resistance. Sp-CCR5-gRNA6^2^ targets *CCR5* but has only a single mismatch in an off-target site in the highly homologous *CCR2* gene (Fig. 2A). This results in approximately similar mutation frequencies of both target *CCR5* and off-target *CCR2* upon transfection of wild-type SpCas9 and Sp-CCR5-gRNA6 into HEK293 cells (Fig. 2B–E, SpCas9/positive control data). This scenario also exemplifies a class of gRNAs with which high-fidelity Cas9 variants cannot be effectively used due to dramatic decreases in on-target activity: though the high-fidelity Cas9 variant eSpCas9(1.1) results in reduced off-target activity at *CCR2* (Supplementary Fig. 1A,B), it simultaneously causes a drastic 12-fold reduction in on-target activity at *CCR5* to nearly undetectable levels (Supplementary Fig. 1C,D), which is in agreement with existing literature for this gRNA^11^.

Instead, we developed an *S. aureus* based PROTECTOR strategy such that the PROTECTOR-gRNA is predicted to be specific to the off-target *CCR2* site: it has 100% sequence identity (except for a 5’ G required for transcription from the U6 promoter) to the off-target site, but has one major mismatch to the target site in *CCR5* that creates a sub-optimal PAM (NGGRRT→ NGGRRG)^28^ as well as another more minor mismatch 16-bp upstream of the PAM (Fig. 2A). Indeed, stable expression of PROTECTOR components (dSaCas9 and Sa-CCR2-gRNA) in advance of transfection of SpCas9 and Sp-CCR5-gRNA6, resulted in a 3.5-fold reduction in off-target activity (*p* < 0.0001) (Fig. 2B,C) and, surprisingly, a 1.2-fold increase in on-target activity (*p* = 0.0007) (Fig. 2D,E) compared to cells stably expressing control PROTECTOR components (dSaCas9 and Sa-gRNAscr). Stable expression of PROTECTOR components had no discernible effects on cell growth (Supplementary Fig. 2). Thus, unlike the high-fidelity Cas9 strategy, the PROTECTOR strategy can reduce off-target activity without compromising on-target efficacy at *CCR5*.

### The PROTECTOR strategy can protect off-target sites that are not solved by the use of high-fidelity Cas9 variants

Nevertheless, high-fidelity Cas9 enzymes can, with other gRNAs, be highly effective at reducing off-target mutagenesis without a reduction in on-target activity. Though dozens of off-target sites are characteristic of wild-type SpCas9 activity, implementation of high-fidelity Cas enzymes often reduces the number of off-target sites to zero or one. We next sought to assess whether the PROTECTOR strategy can be implemented in combination with high-fidelity Cas9 enzymes in order to shield a single off-target site that continues to be edited despite an otherwise effective high-fidelity Cas9 application. This also presents a more practical use of the PROTECTOR strategy in preventing the off-target mutagenesis of a single site, rather than the multiple off-target sites that occur upon wild-type Cas9 activity and would thus necessitate multiple PROTECTOR gRNAs.

A relevant example of this scenario is the FANCF site 2 gRNA: while wild-type SpCas9 has been shown to exhibit activity at 25 off-target sites when used with this gRNA, a high fidelity SpCas9 variant continues to display off-target activity at only the top off-target site, named off-target 1 or “OT1”, at a rate approximately equal to its on-target activity at *FANCF*^4,8^. Our data on the cleavage activity of SpCas9 and the high-fidelity variant eSpCas9(1.1) (Supplementary Fig. 3A–D) corroborates these findings: OT1 is mutated by both SpCas9 and eSpCas9(1.1). Compared to wild-type SpCas9, eSpCas9(1.1) produced only a mild 1.1-fold reduction in off-target activity (51.7 vs. 45.3%, *p* = 0.0126) (Supplementary Fig. 3A,B) and no significant change in on-target activity (48.8% vs. 48.8%) (Supplementary Fig. 3C,D).

**Figure 3:**
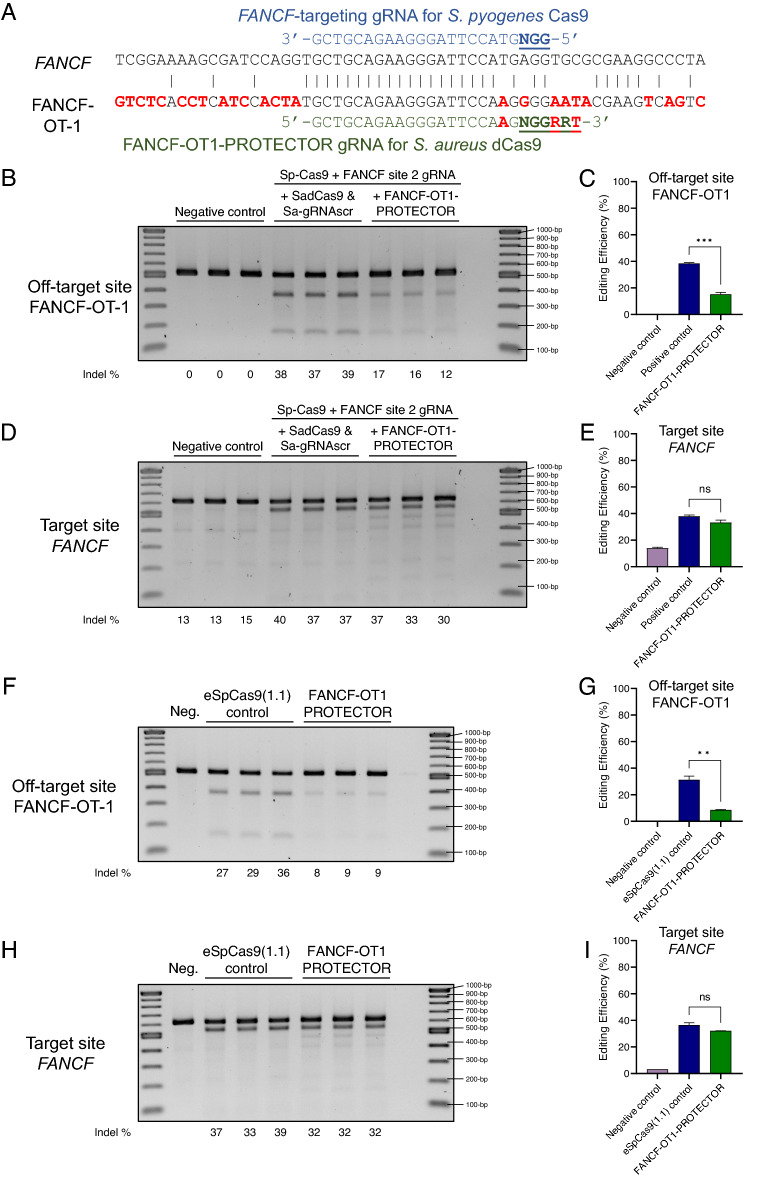
The PROTECTOR strategy applied to HEK293 cells transfected with CRISPR/Cas9 targeting *FANCF* site 2. (**A**) Schematic showing the specificity of the PROTECTOR gRNA for the off-target locus based on the sequence comparison between the target of FANCF site 2 gRNA in the *FANCF* gene and the major off-target locus, OT1. Mismatches in the OT1 sequence are bolded and in red. FANCF site 2 gRNA is depicted in blue with the PAM sequence bolded and underlined. The PROTECTOR gRNA is depicted in green with the PAM sequence bolded and underlined. Mismatches between the PROTECTOR gRNA sequence and the target site sequence are also bolded and in red. (**B**–**E**) depict the use of wild-type Cas9 with and without the PROTECTOR strategy while (**F**–**I**) depict the use of high-fidelity Cas9 variant eSpCas9(1.1) with and without the PROTECTOR strategy. Agarose gel results of T7E1 assay for OT1 and target site in *FANCF* are depicted in (**B**) and (**D**) or (**F**) and (**H**) for wild-type SpCas9 or eSpCas9(1.1), respectively. The first lane contains 1 kb Plus DNA Ladder (NEB), followed by results of T7E1 assays performed on the following biological triplicate sets of transfected samples. Summarized as “Negative control”, HEK293 cells stably expressing dSaCas9 and PROTECTOR gRNA that were transfected with 125 ng SpCas9 plasmid and 125 ng scrambled non-targeting Sp-gRNA plasmid (Sp-gRNAscr). Summarized as "Positive control” in (**C**) and (**E**), HEK293 cells stably expressing dSaCas9 and scrambled non-targeting Sa-gRNA plasmid (Sa-gRNAscr), transfected with 125 ng SpCas9 plasmid and 125 ng *FANCF* site 2 plasmid. Summarized as “FANCF-OT1 PROTECTOR” in (**C**) and (**E**), HEK293 cells stably expressing dSaCas9 and PROTECTOR gRNA, transfected with 125 ng SpCas9 plasmid and 125 ng *FANCF* site 2 plasmid. The final two lanes contain a negative (water) control for the T7E1 assay followed by 1 kb Plus DNA Ladder. (**F**–**I**) are the same as (**B**–**E**) except that they include only a single negative control replicate and wild-type SpCas9 plasmid was replaced with eSpCas9(1.1) plasmid across all samples. Indel estimates for each sample are depicted at the bottom of each lane, rounded to the nearest whole number. The results of (**B**) and (**D**) are quantified in (**C**) and (**E**), respectively, and the results of (**F**) and (**H**) and quantified in (**G**) and (**I**), respecitively. Data in (**C**, **E**, **G**, **I**) are presented as mean ± SEM and were quantified with ImageJ: editing efficiency is calculated as the intensity of T7E1 cleavage products divided by the sum of the intensities of T7E1 cleavage products and the uncut PCR amplicon. Additional bands at approximately 200-bp and 400-bp in all negative control *FANCF* on-target samples are a consequence of a large HEK293-specific insertion within the *FANCF* on-target amplicon and do not reflect gene editing results. These bands–as well as additional bands that reflect digestion products of DNA bearing both this mutation and genome editing outcomes–are also present in FANCF site 2 gRNA conditions and were ignored for all calculations. *** represents *p* < 0.001 (independent t-test) and ns indicates that differences are not statistically significant.

We developed a PROTECTOR-gRNA (Fig. 3A) for the off-target site and first assessed its efficacy when used with wild-type SpCas9. When expressed stably in cells, PROTECTOR components yielded a 2.5-fold reduction in off-target activity compared to controls (*p* = 0.0001) (Fig. 3B,C). Thus, the PROTECTOR strategy alone, unlike the high-fidelity Cas9 variant strategy alone, is a viable solution to off-target mutagenesis at OT1. Notably, similar to eSpCas9(1.1) and unlike the case of *CCR5*, there was no significant increase in on-target activity (Fig. 3D,E).

We then assessed whether the PROTECTOR strategy could be combined with eSpCas9(1.1) to still reduce off-target mutagenesis at OT1. Indeed, combining the FANCF-OT1 PROTECTOR strategy with eSpCas9(1.1) resulted in a 3.4-fold decrease in off-target activity at OT1 (*p* = 0.0011) (Fig. 3F,G) and no significant difference in on-target activity (Fig. 3H,I). These data provide evidence to the viability and potential synergistic benefits of combining high-fidelity nucleases with the PROTECTOR strategy.

### Addition of KRAB domains does not improve PROTECTOR editing outcomes

Krüppel associated box (KRAB) domains are potent transcriptional repressors^29^ which–when fused to dCas9–dramatically improve the efficacy of CRISPR/dCas9-based targeted transcriptional interference systems compared to dCas9 alone^30^. This is thought to partly be a consequence of KRAB-directed heterochromatization^31^ of the target locus, which reduces the accessibility of the DNA to transcription factors; heterochromatization is thought to impair Cas9 activity in the same manner^32,33^. We thus hypothesized that the efficacy of the PROTECTOR strategy could be augmented by the fusion of a KRAB domain to dSaCas9.

To assess this possibility, we repeated the above experiments with one modification to the PROTECTOR strategy: the dSaCas9 plasmid was replaced with one encoding dSaCas9-KRAB. We found that stable expression of dSaCas9-KRAB instead of dSaCas9 provided no apparent benefit: while dSaCas9-KRAB succeeded in producing 2.72-fold (*p* < 0.0001) and 2.36-fold (*p* < 0.0001) reductions in off-target activity at *FANCF* and *CCR5* off-target sites, respectively, compared to controls, it also resulted in 1.19-fold (*p* = 0.0008) and 2.28-fold (*p* = 0.002) reductions in on-target activity, respectively (Supplementary Fig. 4).

### Co-delivery of PROTECTOR components with active CRISPR/Cas9 successfully reduces off-target editing

In the previous experiments, PROTECTOR components were stably expressed prior to transfection of active Cas9 and gRNA to ensure that all transfected cells also expressed PROTECTOR components. As this is not a favorable clinical strategy, we sought to determine whether PROTECTOR can retain its efficacy when it is co-delivered with active CRISPR/Cas9 components. To maximize the likelihood of PROTECTOR co-delivery into cells receiving active Cas9 components, we used a 10:1 stoichiometric of PROTECTOR to active Cas9 components. We also tested the efficacy of two different strategies wherein PROTECTOR components were either first transfected into cells 24 h prior to transfection of active Cas9 components (“pre-transfection”) or simultaneously co-transfected alongside active Cas9 and gRNA plasmids (“co-transfection”) (Supplementary Fig. 5A). To our surprise, while pre-transfection did not result in any significant change in off-target editing at FANCF-OT1, co-transfection of PROTECTOR produced a 2.34-fold (*p* = 0.0092) reduction in off-target editing compared to cells transfected with control PROTECTOR components (dSaCas9 and Sa-gRNAscr) (Supplementary Fig. 5A,B). These data suggest that simultaneous co-delivery of PROTECTOR components alongside active Cas9 components can reduce off-target editing.

## Discussion

In this work, we demonstrate that the PROTECTOR strategy–which invokes a dCas9 protein orthologous to a Cas9 protein used for gene editing–can specifically reduce off-target effects of CRISPR/Cas9 gene editing at previously determined off-target sites. We show further that the PROTECTOR strategy can complement high-fidelity Cas variants to produce even more favorable gene editing outcomes, such as the combined use of eSpCas9(1.1)–which has been shown to eliminate all off-target sites of the FANCF site 2 gRNA except for OT1–and PROTECTOR–which specifically reduces off-target effects at OT1. We also find that the addition of a KRAB domain does not improve the PROTECTOR strategy and that co-transfection of PROTECTOR with active CRISPR/Cas9 components can be an effective strategy to reduce off-target editing; to our surprise, co-transfection was more efficient than pre-transfection conditions in which PROTECTOR components were delivered to cells 24 h before active CRISPR/Cas9 components. This may be a consequence of the fact that the pre-transfection strategy might result in transfection of different populations of cells with PROTECTOR or active components–especially in the context of actively dividing cells–whereas the co-transfection condition transfects the same cells with all components and ensures that PROTECTOR components are present in cells that receive active CRISPR/Cas9.

A major limitation of the PROTECTOR strategy is its laboriousness: it requires previously determined off-target sites compounded with a likely low upper limit for the number of such off-target sites that can be blocked in any given cell as well as a need for well-developed specific ortholog strategies and gRNAs with PAMs at these off-target sites. These requirements render PROTECTOR largely impractical for most laboratory research cases involving gene knockout. However, in clinical cases, where a specific single gRNA is developed to treat a particular condition, the process of defining the gRNA’s off-targets should be routine and the development of a specific PROTECTOR strategy not particularly difficult.

Though CRISPR/Cas9 can be multiplexed with multiple gRNAs^34^–including one study that reported the use of 12 gRNAs with high efficiencies of each^35^–it is yet unclear whether PROTECTOR can be applied in the same manner with high efficiency across multiple off-target sites. Still, the need to apply the PROTECTOR strategy to multiple off-target sites may be limited: this is demonstrated by the example of the FANCF site 2 gRNA when used in combination with the high-fidelity variant eSpCas9(1.1). Here, while 25 off-target sites are detected when wild-type SpCas9 is employed, there is only detectable off-target activity at a single site when high-fidelity SpCas9 is used in place of wild-type SpCas9^4^. Therefore, when high-fidelity SpCas9 is used, only a single off-target site (FANCF-OT1) remains to be addressed by additional off-target reduction strategies. We show in this work that indeed the PROTECTOR strategy reduces off-target mutagenesis at this single off-target site when used in tandem with a high-fidelity SpCas9 variant.

There are a number of additional limitations inherent to the experiments performed herein to demonstrate the PROTECTOR strategy. The results of the human cell experiments in this study are limited by the use of the T7E1 assay to assess mutation rates. This assay may not accurately reflect true mutation rates, particularly because it has reduced sensitivity for short mutations^36^. Further research is necessary to determine the impact of PROTECTOR on both target and off-target mutation rates with greater precision. Furthermore, the purpose of the experiments described herein was to determine whether orthologous nuclease-dead Cas9 can be targeted to off-target sties in order to reduce off-target mutagenesis. In these experiments, PROTECTOR components were given clear advantage over catalytically active components by either excess quantities in vitro (~ 10:1 stoichiometric ratio PROTECTOR:catalytically active components) or, in human cells, by stable expression of PROTECTOR components prior to transfection of catalytically active components. Co-delivery by co-transfection of PROTECTOR and active CRISPR/Cas9 components was indeed successful in reducing off-target editing, yet a 10:1 stoichiometric ratio was still used. Finally, in these experiments, gene editing at the off-target sites was reduced by PROTECTOR but was never fully eliminated, a fact that could potentially be resolved in the future by extensive optimization. There remains considerable work to be done to determine whether the PROTECTOR strategy would prove successful upon delivery of both PROTECTOR and catalytically active components by clinically relevant techniques and at stoichiometric ratios that provide clinically relevant efficacy of both CRISPR/Cas9 and PROTECTOR components.

The rationale behind PROTECTOR–inhibition of Cas9 activity by competitive binding of a catalytically dead complex to off-target sites–has been previously developed into a technique by two separate groups^9,10^. However, both groups used a different strategy than the one presented herein, instead introducing short gRNAs that direct the binding of Cas9 to off-target sites but are too short to stimulate cleavage by Cas9, resulting in the binding of a dead complex that competes with active Cas9. In addition to potentially being applicable in specific cases where the truncated gRNA strategies fail, the PROTECTOR strategy, in principle, offers a completely separate capacity. Since truncated gRNAs still direct the binding of Cas9 protein, they are not suitable in applications where binding by a dCas9 protein is the mode of editing: this includes some forms of base editing–in which dCas9 is fused to a deaminase to produce site-specific DNA base changes^37^–and epigenetic editing–in which dCas9 is fused to any of a large number of protein domains with epigenetic-modifying capacity, such as p300, KRAB, or DNMT3A^38^. On the other hand, in the PROTECTOR strategy, an orthologous dCas protein should in theory still interfere with the binding of base editors and epigenetic editors, though these applications need to be addressed with future experiments.

We further recognize that there is a need to determine the optimal stoichiometric ratios of catalytic CRISPR/Cas components to PROTECTOR components, which is expected to exhibit high variability on a target-to-target basis as well as with mode of delivery, target cell type, and Cas species selection. While we have shown that co-transfection of PROTECTOR with active Cas9 is effective in reducing off-target editing, it will be essential to carefully optimize PROTECTOR stoichiometry and delivery in the context of specific gRNAs and their associated delivery methods. We therefore hope that groups with prioritized candidate gRNAs with clinically relevant delivery methods (e.g., nanoparticle-based delivery of ribonucleoproteins) to specific target cells will find this new method useful as a starting point for optimization for specific cases so that the PROTECTOR strategy might become a valuable complement to the existing suite of tools that reduce off-target CRISPR/Cas effects in order to facilitate the safer genome editing that is necessary for the clinical setting.

## Methods

### In vitro transcription of gRNAs

In vitro transcription of gRNAs was performed as described previously^18,39^ and according to version 11 of the associated online protocol (dx.doi.org/10. 17504/protocols.io.bfxfjpjn). Briefly, variable oligonucleotides (T7FVar) (Supplementary Table 1) encoding the T7 promoter and custom gRNA sequence were annealed to partially complementary oligonucleotides (T7R_Long) encoding gRNA scaffold and transcriptional terminator sequences and amplified using Phusion polymerase (Thermo Fisher). As these protocols were designed for *S. pyogenes*, we also developed modified sequences to replace the scaffold with the sequence required for *S. aureus* gRNAs as well as modified primers for amplification (Supplementary Table 1). 8 µL of the PCR reaction was directly used as DNA template for in vitro transcription with the HiScribe T7 High Yield RNA Synthesis Kit (NEB) according to the manufacturer’s protocol. The in vitro transcribed RNA was then treated with 1 µL DNase (NEB) for 15 min at 37 °C to remove the template DNA and 5’-triphosphates were removed by incubation for 3 h at 37 °C with 20 units Quick CIP (NEB) in 1X CutSmart buffer in a final volume of 100 µL. Finally, the gRNAs were purified using the miRNeasy Micro Kit (Qiagen) according to the manufacturer’s protocol and eluted in 30 µL nuclease-free water. Final gRNA concentrations were determined using the Qubit RNA Broad Range Assay Kit (Thermo Fisher).

### In vitro PROTECTOR assay

The piggyBac plasmid used for in the in vitro PROTECTOR assay was obtained from Addgene (plasmid #97421). Recombinant SpCas9 was purchased from NEB (catalog no. M0386S). and recombinant dSaCas9 was purchased from Applied Biological Materials (catalog no. K046). PROTECTOR ribonucleoprotein complexes (RNPs) were first formed by incubating 8 µL of 1 µM stock dSaCas9 protein with 3 µL of 3 µM stock in vitro transcribed Sa-gRNAscr or Sa-PROTECTOR gRNA with 3 µL 10X Cas9 reaction buffer (Applied Biological Materials) and 10 µL nuclease-free water for 10 min at room temperature. 6 µL of 30 nM stock piggyBac plasmid was added to this reaction, which was then incubated for an additional 1 h at 37 °C to facilitate RNP binding to the target DNA. 15 min before this reaction finished, SpCas9 RNPs were separately prepared by mixing 23 µL nuclease-free water, 3 µL 10X Cas9 reaction buffer, 3 µL 300 nM stock Sp-gRNA (targeting two sites in piggyBac vector), and 1 µL 1 µM stock SpCas9 and incubating the reaction for 10 min at room temperature. When both dSaCas9 and SpCas9 reactions were finished, they were mixed together (60 µL final volume) and incubated for an additional 15 min at 37 °C. Then we added 2 µL of 20 mg/mL stock Proteinase K solution and continued to incubate the reaction for 15 min at 37 °C followed by 30 min at 55 °C. DNA was re-purified with the Monarch PCR & DNA Cleanup Kit (NEB) according to the manufacturer’s protocol and eluted in 20 µL nuclease-free water. We then added 4 µL 6X DNA loading dye (Thermo Fisher) and ran the samples on a 1% agarose gel with 7–10 µL GeneRuler 1 kb DNA ladder (Thermo Fisher).

### Plasmids

All *S. pyogenes* gRNA expression plasmids were generated by mutagenesis of pLenti-gRNA-puro (Addgene plasmid #180426) with the Q5® Site-Directed Mutagenesis Kit as previously described^40^ using primers listed in Supplementary Table 1. A modified species-specific pLenti-gRNA-puro plasmid for *S. aureus* gRNAs was constructed as described previously for *S. pyogenes*^18^ by ordering modified gene fragments (gBlocks, Integrated DNA Technologies) encoding the U6 promoter, gRNA sequence, and species-specific scaffold (Supplementary Table 1). Briefly, the gBlock was resuspended and amplified in a standard Taq polymerase (Thermo Fisher) reaction using gBlock_gRNA_F/R primers (Supplementary Table 1) and subcloned into pCR2.1-TOPO using the TOPO™ TA Cloning™ Kit (Thermo Fisher) according to the manufacturer’s protocol. This plasmid was then digested with EcoRI and cloned into pLenti-gRNA-puro (digested with EcoRI) to directly replace the *S. pyogenes* gRNA and scaffold. The resulting plasmid was used as a template for synthesis of all additional gRNA sequences by mutagenesis as described above for pLenti-gRNA-puro using primers listed in Supplementary Table 1. This plasmid is available on Addgene as pLenti-Sa-gRNA-puro (Addgene plasmid #191652). Wild-type SpCas9 (lenti-Cas9-Blast) and eSpCas9(1.1) plasmids were obtained from Addgene (plasmid #52962 and #138555). The plasmid encoding lentiviral dSaCas9 was constructed by cloning Sa-dCas9-NLS-3xFLAG/pcDNA3.1 (Addgene plasmid #98041) into pLenti- V6.3 Ultra (Addgene plasmid #106172) using the restriction enzymes NheI-HF and EcoRI-HF followed by ligation with T4 ligase (NEB) and is now available on Addgene as pLenti-eGFP-dSaCas9-blast (Addgene plasmid #191653). Lentiviral plasmid encoding dSaCas9-KRAB was produced by cloning dSaCas9-KRAB from the plasmid AAV CMV-dSaCas9-KRAB-bGHpA (Addgene plasmid #106219) using NdeI and EcoRI-HF (NEB) into pLenti- V6.3 Ultra and is now available on Addgene as pLenti-dSaCas9-KRAB (Addgene plasmid #191654).

### Cell culture experiments

HEK293 and HEK293T cell lines were purchased from ATCC (catalog no. CRL-1573 and CRL-3216). Cells were grown in a humidified 5% CO_2_ 37 °C incubator in Gibco DMEM, high glucose medium (Thermo Fisher) supplemented with 10% fetal bovine serum and 1X final concentration of 100X penicillin–streptomycin-glutamine (Thermo Fisher). To produce HEK293 cells stably expressing PROTECTOR gRNA and dSaCas9, lentiviral particles were first produced in HEK293T cells. 3.75 × 10^6^ HEK293T cells were plated on 100-mm tissue-culture dishes. After 24 h, cells were transfected using X-tremeGENE 9 DNA Transfection Reagent (Millipore Sigma) according to the manufacturer’s protocol: 1.3 pmol psPAX2 (Addgene plasmid #12260), 0.72 pmol pMD2.G (Addgene plasmid #12259), and 1.64 pmol of pLenti-eGFP-dSaCas9 were mixed in 100 µL Opti-MEM medium (Thermo Fisher) in a 1.5 mL tube. In a separate 1.5 mL tube, X-tremeGENE 9 was mixed into 500 µL Opti-MEM medium at a ratio of 3:1 (X-tremeGENE 9 in µL : total plasmid amount in µg). The two mixes were combined, incubated at room temperature for 20 min, and then added to the plated HEK293T cells in a drop-wise manner. 48 h later, viral supernatant was collected by filtering through a 0.45 µm PVDF syringe filter unit (Millipore Sigma) and concentrated tenfold (1 mL final volume) using the Lenti-X Concentrator (Takara) according to the manufacturer’s protocol. For viral transduction, 100,000 HEK293 cells were plated 24 h in advance in 6-well tissue-culture dishes, then the medium was aspirated and replaced with 1 mL concentrated viral supernatant, replaced again with complete DMEM 24 h later, and replaced again 24 h later with DMEM containing 10 µg/mL blasticidin. The antibiotic-containing medium was replenished every 48 h until selection was complete and all non-transduced control cells had died. The same process was then repeated to produce viral particles for each lentiviral Sa-gRNA plasmid, which were used to infect the already established HEK293-dSaCas9 cells followed by selection with 2 µg/mL puromycin. For cleavage activity assays, active SpCas9, eSpCas9(1.1) and Sp-gRNA plasmids (according to the amounts specified in figure legends) were then transfected with a 3:1 ratio of Xtreme-GENE 9 as described above into HEK293 cells (100,000 plated in 6-well tissue-culture dishes 24 h in advance). For co-transfection experiments in Supplementary Fig. 5, the transfected quantities were: 50 ng SpCas9 plasmid, 50 ng FANCF site 2 gRNA plasmid, 500 ng dSaCas9 plasmid, and 500 ng scrambled control or OT1-PROTECTOR gRNA plasmid complexed with 3.3 µL X-tremeGENE 9. In pre-transfection experiments in the same figure, PROTECTOR/control was first transfected alone (500 ng dSaCas9 and 500 ng control or OT1-PROTECTOR gRNA with 3 µL X-tremeGENE 9) 24 h after plating the cells, and then active Cas plasmids (SpCas9 and FANCF site 2 gRNA, 50 ng each) were transfected 24 h later. To test whether stable expression of PROTECTOR components affected cell growth, cell lines were plated at 50,000 cells per well–in triplicates per time point–of a 6-well tissue culture dish. To count only viable cells, trypsinized cell suspensions were mixed 1:1 with Trypan Blue 0.4% solution (Thermo Fisher) and incubated for 1 min prior to counting. Cells were counted manually using a hematocytometer 24 h, 48 h, 72 h, and 96 h after plating.

### DNA Isolation and T7E1 assay

48 h after transfection with catalytically active CRISPR/Cas9 components, genomic DNA was collected by removing the medium and replacing with 400 µL DNA lysis buffer (100 mM Tris, pH 7.5, 150 mM NaCl, 0.5% SDS, 10 mM EDTA). The solution was incubated with 1 µL 20 mg/mL RNAse A (NEB) for 30 min at 37 °C followed by the addition of 5 µL 20 mg/mL Proteinase K (Sigma) and incubation for 2 h at 55 °C. DNA was purified by the addition of 200 µL phenol solution and 200 µL chloroform, mixing, centrifugation at 16,000 × g for 5 min at 4 °C, transfer of the aqueous phase to a new 1.5 mL tube, addition of 400 µL chloroform, mixing, centrifugation at 16,000 × g for 5 min at 4 °C, transfer of the aqueous phase to a new 1.5 mL tube, and precipitation by the addition of 1 mL ice-cold 95% ethanol and incubation for 30 min at -80 °C. DNA was pelleted by centrifugation at 16,000 × g for 15 min at 4 °C, washed one time with 750 µL 70% ethanol, and centrifuged at 16,000 × g for 5 min at 4 °C. DNA was then air dried for 5 min and resuspended in 100 µL nuclease-free water. DNA concentration was measured with a NanoDrop 2000 spectrophotometer and diluted to 100 ng/µL. T7E1 assays were performed with the EnGen® Mutation Detection Kit (NEB) according to the manufacturer’s protocol. For each sample, 1 µL of 100 ng/µL DNA solution was used as input for the first step (PCR) of the T7E1 workflow. PCR primers, annealing temperatures, and band sizes are listed in (Supplementary Table 2). After completion of the T7E1 digestion reaction, 4 μL of Gel Loading Dye, Purple (6X) (NEB) was added to the reaction and the entire volume was loaded on a 2% agarose gel alongside 7 µL 1 kb Plus DNA Ladder (NEB) and run at 80 V for 20 min followed by 100 V for 1 h. The gel was stained in 100 mL deionized (DI) water containing 20 µL of 10 mg/mL ethidium bromide on a rocker for 15 min, washed in DI water, de-stained on a rocker for 15 min in DI water, and then imaged on a Molecular Imager Gel Doc XR + System (Bio-Rad). Band intensities were quantified using ImageJ according to a previously described protocol^41^. Percent editing efficiencies were calculated as the summed intensities of the two T7E1 cleavage products divided by the summed intensities of the two T7E1 cleavage products and the intensity of the uncut PCR band. Statistical tests were performed with GraphPad Prism 9.

## Supplementary Information

Supplementary Information.

## Data Availability

All data generated or analyzed during this study are included in this published article and its supplementary information files.

## References

[1] Fu Y (2013). High-frequency off-target mutagenesis induced by CRISPR-Cas nucleases in human cells. Nat. Biotechnol..

[2] Cho SW (2014). Analysis of off-target effects of CRISPR/Cas-derived RNA-guided endonucleases and nickases. Genome Res..

[3] Naeem M, Majeed S, Hoque MZ, Ahmad I (2020). Latest developed strategies to minimize the off-target effects in CRISPR-cas-mediated genome editing. Cells.

[4] Kleinstiver BP (2016). High-fidelity CRISPR-Cas9 nucleases with no detectable genome-wide off-target effects. Nature.

[5] Slaymaker IM (2016). Rationally engineered Cas9 nucleases with improved specificity. Science.

[6] Schmid-Burgk JL (2020). Highly parallel profiling of cas9 variant specificity. Mol. Cell.

[7] Lee JK (2018). Directed evolution of CRISPR-Cas9 to increase its specificity. Nat. Commun..

[8] Chen JS (2017). Enhanced proofreading governs CRISPR-Cas9 targeting accuracy. Nature.

[9] Coelho MA (2020). CRISPR GUARD protects off-target sites from Cas9 nuclease activity using short guide RNAs. Nat. Commun..

[10] Rose JC (2020). Suppression of unwanted CRISPR-Cas9 editing by co-administration of catalytically inactivating truncated guide RNAs. Nat. Commun..

[11] Liu MS (2020). Engineered CRISPR/Cas9 enzymes improve discrimination by slowing DNA cleavage to allow release of off-target DNA. Nat. Commun..

[12] Kim N (2020). Prediction of the sequence-specific cleavage activity of Cas9 variants. Nat. Biotechnol..

[13] Fu Y, Sander JD, Reyon D, Cascio VM, Joung JK (2014). Improving CRISPR-Cas nuclease specificity using truncated guide RNAs. Nat. Biotechnol..

[14] Murugan K, Suresh SK, Seetharam AS, Severin AJ, Sashital DG (2021). Systematic in vitro specificity profiling reveals nicking defects in natural and engineered CRISPR-Cas9 variants. Nucleic Acids Res..

[15] Jones SK (2021). Massively parallel kinetic profiling of natural and engineered CRISPR nucleases. Nat. Biotechnol..

[16] Sternberg SH, Redding S, Jinek M, Greene EC, Doudna JA (2014). DNA interrogation by the CRISPR RNA-guided endonuclease Cas9. Nature.

[17] Qi LS (2013). Repurposing CRISPR as an RNA-guided platform for sequence-specific control of gene expression. Cell.

[18] Sapozhnikov DM, Szyf M (2021). Unraveling the functional role of DNA demethylation at specific promoters by targeted steric blockage of DNA methyltransferase with CRISPR/dCas9. Nat. Commun..

[19] Tsai SQ (2015). GUIDE-seq enables genome-wide profiling of off-target cleavage by CRISPR-Cas nucleases. Nat. Biotechnol..

[20] Takasugi PR (2022). Orthogonal CRISPR-Cas tools for genome editing, inhibition, and CRISPR recording in zebrafish embryos. Genetics.

[21] Wu X (2014). Genome-wide binding of the CRISPR endonuclease Cas9 in mammalian cells. Nat. Biotechnol..

[22] Gasiunas G (2020). A catalogue of biochemically diverse CRISPR-Cas9 orthologs. Nat. Commun..

[23] Collias D, Beisel CL (2021). CRISPR technologies and the search for the PAM-free nuclease. Nat Commun.

[24] Nishimasu H (2018). Engineered CRISPR-Cas9 nuclease with expanded targeting space. Science.

[25] Walton RT, Christie KA, Whittaker MN, Kleinstiver BP (2020). Unconstrained genome targeting with near-PAMless engineered CRISPR-Cas9 variants. Science.

[26] Ran FA (2015). In vivo genome editing using *Staphylococcus aureus* Cas9. Nature.

[27] Ding S (2005). Efficient transposition of the piggyBac (PB) transposon in mammalian cells and mice. Cell.

[28] Kleinstiver BP (2015). Broadening the targeting range of *Staphylococcus aureus* CRISPR-Cas9 by modifying PAM recognition. Nat. Biotechnol..

[29] Margolin JF (1994). Kruppel-associated boxes are potent transcriptional repression domains. Proc. Natl. Acad. Sci. USA.

[30] Gilbert LA (2013). CRISPR-mediated modular RNA-guided regulation of transcription in eukaryotes. Cell.

[31] Groner AC (2010). KRAB-zinc finger proteins and KAP1 can mediate long-range transcriptional repression through heterochromatin spreading. PLoS Genet..

[32] Kallimasioti-Pazi EM (2018). Heterochromatin delays CRISPR-Cas9 mutagenesis but does not influence the outcome of mutagenic DNA repair. PLoS Biol..

[33] Jain S (2021). TALEN outperforms Cas9 in editing heterochromatin target sites. Nat. Commun..

[34] McCarty NS, Graham AE, Studena L, Ledesma-Amaro R (2020). Multiplexed CRISPR technologies for gene editing and transcriptional regulation. Nat. Commun..

[35] McCarty NS, Shaw WM, Ellis T, Ledesma-Amaro R (2019). Rapid assembly of gRNA arrays via modular cloning in yeast. ACS Synth. Biol..

[36] Sentmanat MF, Peters ST, Florian CP, Connelly JP, Pruett-Miller SM (2018). A survey of validation strategies for CRISPR-Cas9 editing. Sci. Rep..

[37] Komor AC, Kim YB, Packer MS, Zuris JA, Liu DR (2016). Programmable editing of a target base in genomic DNA without double-stranded DNA cleavage. Nature.

[38] Nakamura M, Gao Y, Dominguez AA, Qi LS (2021). CRISPR technologies for precise epigenome editing. Nat. Cell Biol..

[39] DeWitt MA (2016). Selection-free genome editing of the sickle mutation in human adult hematopoietic stem/progenitor cells. Sci. Transl. Med..

[40] Sapozhnikov DM, Szyf M (2022). Enzyme-free targeted DNA demethylation using CRISPR-dCas9-based steric hindrance to identify DNA methylation marks causal to altered gene expression. Nat. Protoc..

[41] Stael S, Miller LP, Fernandez-Fernandez AD, Van Breusegem F (2022). Detection of damage-activated metacaspase activity by western blot in plants. Methods Mol. Biol..

