# Supplementary material for: The PROTECTOR strategy employs dCas orthologs to sterically shield off-target sites from CRISPR/Cas activity
Source: Sci Rep. 2023 Feb 9;13:2280. doi: 10.1038/s41598-023-29332-2 (PMC9911626; doi:10.1038/s41598-023-29332-2)

Supplementary Information

for

The PROTECTOR strategy employs dCas orthologs to sterically shield off-target sites from CRISPR/Cas activity

Daniel M. Sapozhnikov and Moshe Szyf

The Supplementary Information file contains Supplementary Figures 1-4, Supplementary Tables 1-2, and uncropped versions of agarose gels presented in the main and supplementary figures.

Supplementary Figure 1

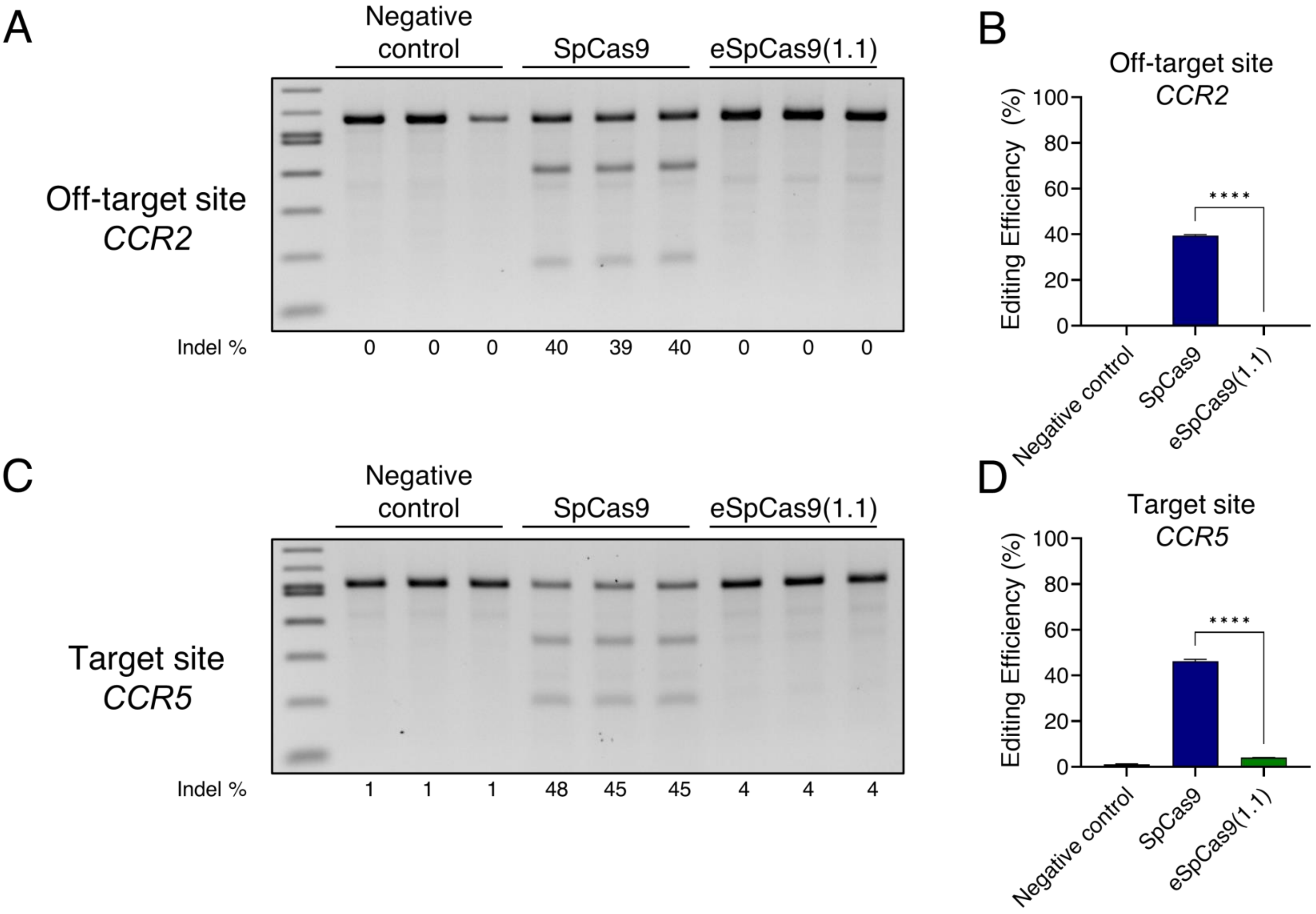

Supplementary Figure 1. Efficacy and specificity of high-fidelity Cas enzyme eSpCas9(1.1) compared to wild-type SpCas9 at CCR5 and CCR2. Agarose gel results and quantifications of T7E1 assays in experimental triplicates for CCR2 in (A) and (B), and CCR5 in (C) and (D). Negative control: control HEK293 cells transfected with 250ng wild-type SpCas9 plasmid and 250 ng scrambled non-targeting Sp-gRNA. SpCas9: control HEK293 cells transfected with 250 ng wild-type SpCas9 plasmid and 250 ng Sp-CCR5-gRNA6 plasmid. eSpCas9(1.1): control HEK293 cells transfected with 250ng eSpCas9(1.1) plasmid and 250ng Sp-CCR5-gRNA6 plasmid. Indel frequency estimates derived from the T7E1 assay for each sample are depicted at the bottom of each lane, rounded to the nearest whole number. The abbreviation “Neg.” in the bar charts corresponds to “Negative control” in the agarose gel results. Data in (B) and (D) are presented as mean ± SEM and were quantified with ImageJ: editing efficiency is calculated as the intensity of T7E1 cleavage products divided by the sum of the intensities of T7E1 cleavage products and the uncut PCR amplicon. * indicates $p<0.05$, **** indicates $p<0.0001$, and ns indicates no statistically significant difference by independent t-test.

Supplementary Figure 2

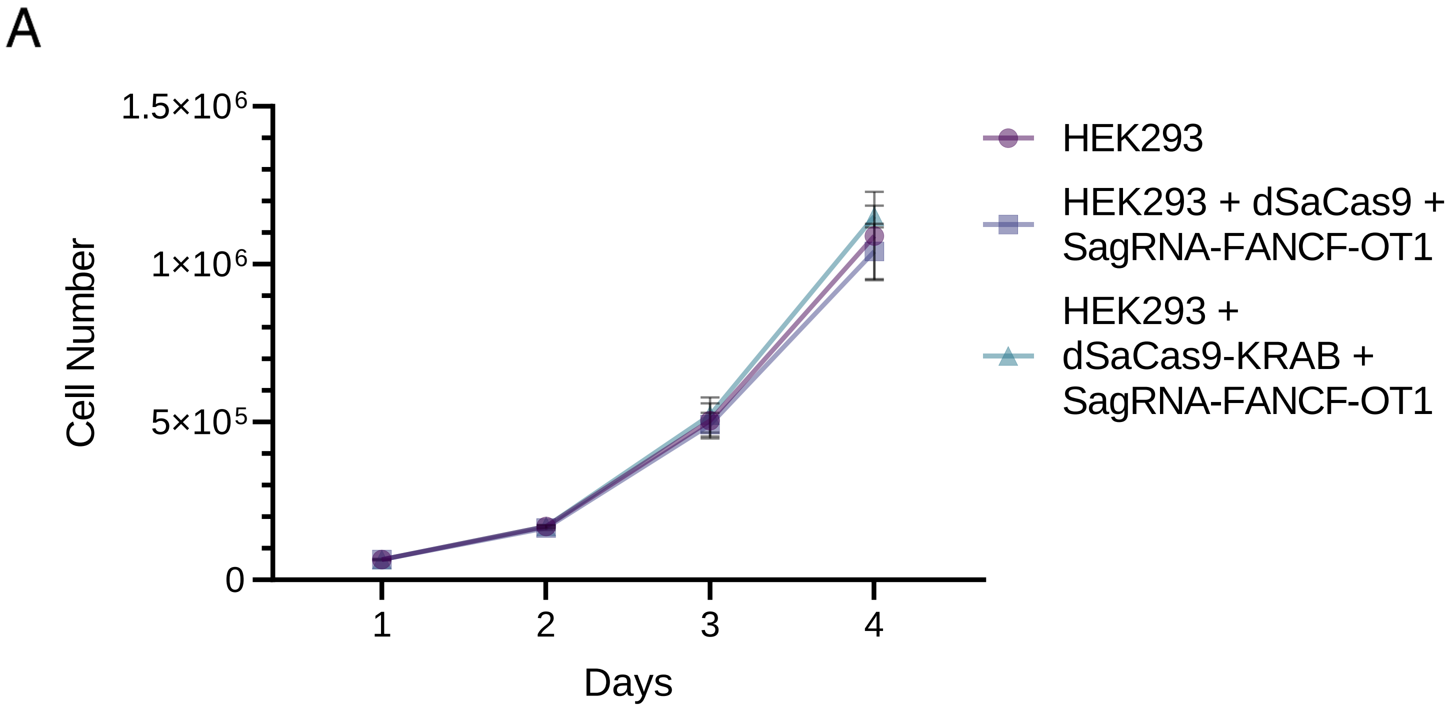

Supplementary Figure 2. Stable expression of PROTECTOR components does not affect cell growth. (A) Viable cell count over four days of cell line expansion in culture as measured with the trypan blue exclusion test and counted manually with a hematocytometer. HEK293 indicates a control cell line with no modifications. HEK293 + dSaCas9 + SagRNA-FANCF-OT1 are HEK293 cells that stably express both PROTECTOR components used in experiments to reduce off-target mutagenesis of SpCas9 when used with FANCF site 2 gRNA; HEK293 + dSaCas9-KRAB + SagRNA-FANCF-OT1 are the same but dSaCas9 is fused to a KRAB repressive domain as used for experiments presented in Supplementary Figure 4. Data is presented as mean ± SEM total cell number per well.

Supplementary Figure 3

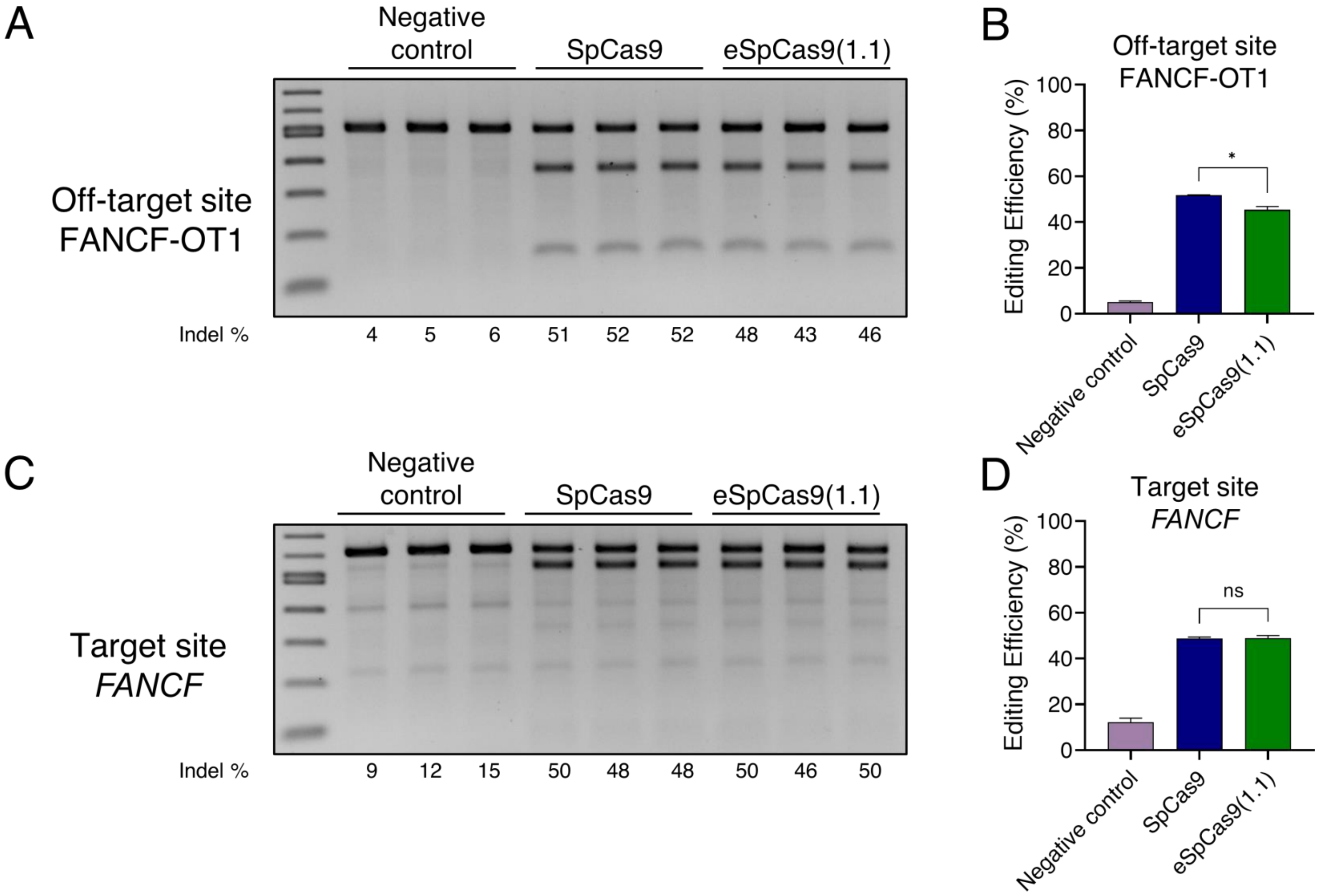

Supplementary Figure 3. Efficacy and specificity of high-fidelity Cas enzyme eSpCas9(1.1) compared to wild-type SpCas9 at FANCF and the primary off-target site of FANCF site 2 gRNA, FANCF OT1. Agarose gel results and quantifications of T7E1 assays in experimental triplicates for FANCF-OT1 in (A) and (B), and FANCF in (C) and (D). Negative control: control HEK293 cells transfected with 250ng wild-type SpCas9 plasmid and 250 ng scrambled non-targeting Sp-gRNA. SpCas9: control HEK293 cells transfected with 250 ng wild-type SpCas9 plasmid and 250ng FANCF site 2 gRNA plasmid. eSpCas9(1.1): control HEK293 cells transfected with 250 ng eSpCas9(1.1) plasmid and 250 ng FANCF site 2 gRNA plasmid. Indel frequency estimates derived from the T7E1 assay for each sample are depicted at the bottom of each lane, rounded to the nearest whole number. The abbreviation “Neg.” in the bar charts corresponds to “Negative control” in the agarose gel results. Additional bands at approximately 200-bp and 400-bp in all negative control FANCF on-target samples are a consequence of a large HEK293-specific insertion within the FANCF on-target amplicon and do not reflect gene editing results. These bands – as well as additional bands that reflect digestion products of DNA bearing both this mutation and genome editing outcomes – are also present in FANCF site 2 gRNA conditions and were ignored for all calculations. Data in (B) and (D) are presented as mean ± SEM and were quantified with ImageJ: editing efficiency is calculated as the intensity of T7E1 cleavage products divided by the sum of the intensities of T7E1 cleavage products and the uncut PCR amplicon. * indicates $p<0.05$, **** indicates $p<0.0001$, and ns indicates no statistically significant difference by independent t-test.

Supplementary Figure 4

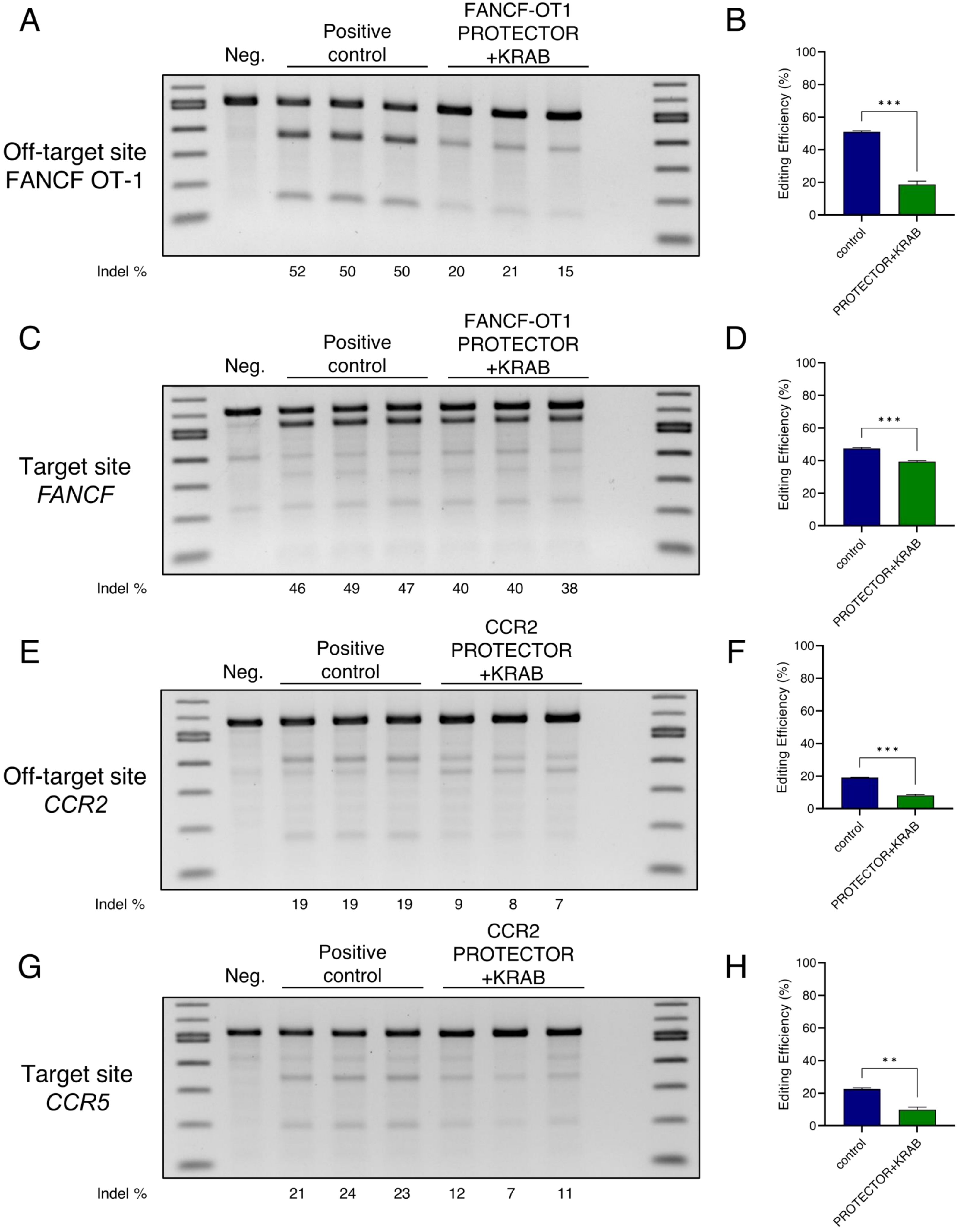

Supplementary Figure 4. Addition of KRAB domain to dSaCas9 does not improve PROTECTOR editing outcomes. Agarose gel results and quantifications of T7E1 assays in experimental triplicates for FANCF OT-1 in (A) and (B), FANCF in (C) and (D), CCR2 in (E) and (F), and CCR5 in (G) and (H). Neg. indicates negative control: HEK293 stably expressing dSaCas9-KRAB and Sa-gRNA scrambled, transfected with 250ng wild-type SpCas9 plasmid and 250ng scrambled non-targeting Sp-gRNA. Positive control: HEK293 stably expressing dSaCas9-KRAB and Sa-gRNA scrambled, transfected with 250ng wild-type SpCas9 plasmid and 250ng FANCF site 2 gRNA (A-D) or Sp-CCR5-gRNA6 (E-H). Positive control: HEK293 stably expressing dSaCas9-KRAB and Sa-gRNA scrambled, transfected with 250ng wild-type SpCas9 plasmid and 250ng FANCF site 2 gRNA (A-D) or Sp-CCR5-gRNA6 (E-H). Indel estimates for each sample are depicted at the bottom of each lane, rounded to the nearest whole number. Additional bands at approximately 200-bp and 400-bp in all negative control FANCF on-target samples are a consequence of a large HEK293-specific insertion within the FANCF on-target amplicon and do not reflect gene editing results. These bands – as well as additional bands that reflect digestion products of DNA bearing both this mutation and genome editing outcomes – are also present in FANCF site 2 gRNA conditions and were ignored for all calculations. Data in (B), (D), (F), and (H) are presented as mean ± SEM and were quantified with ImageJ: editing efficiency is calculated as the intensity of T7E1 cleavage products divided by the sum of the intensities of T7E1 cleavage products and the uncut PCR amplicon. * indicates $p<0.05$, **** indicates $p<0.0001$, and ns indicates no statistically significant difference by independent t-test.

Supplementary Figure 5

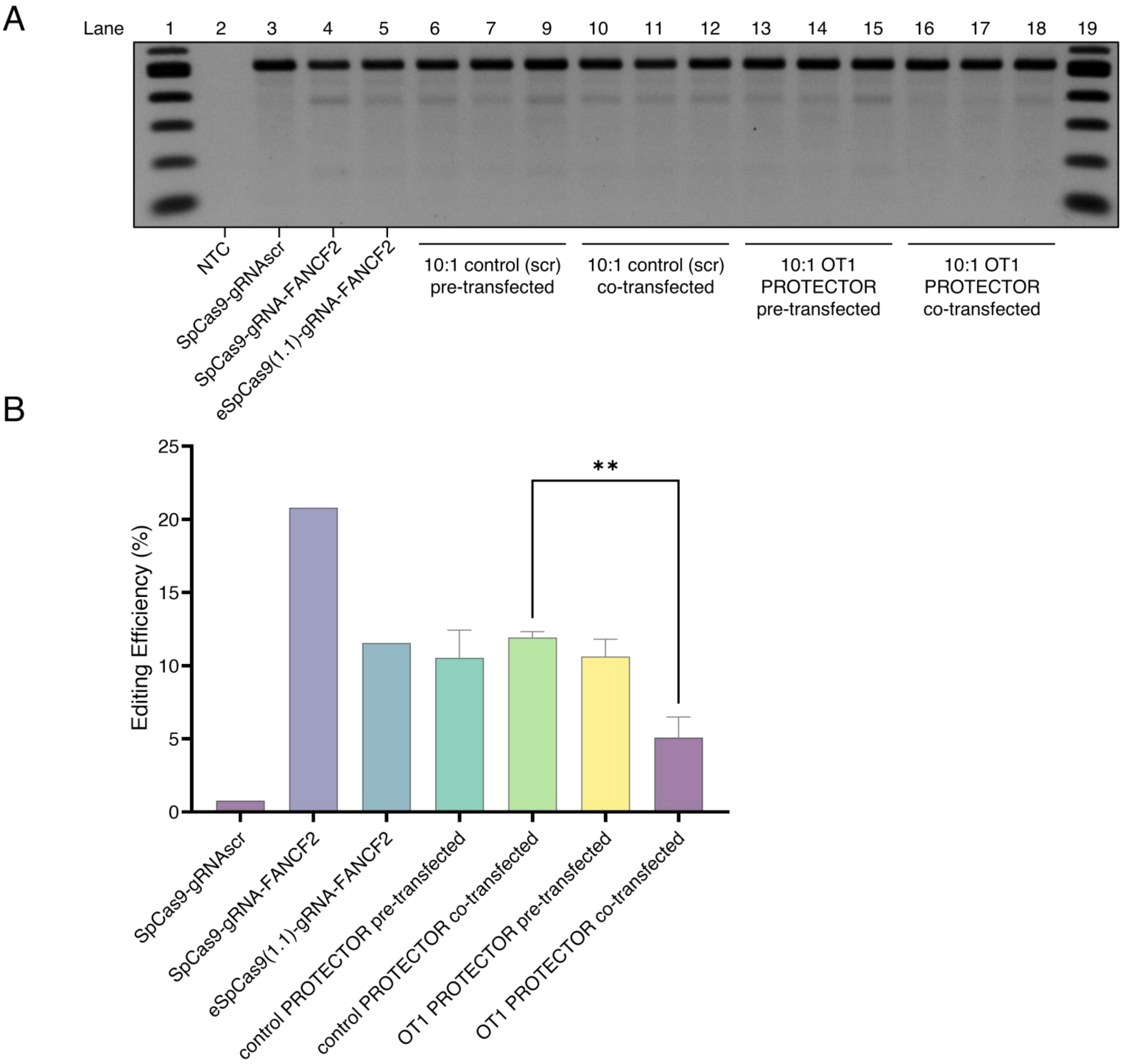

Supplementary Figure 5. Assessment of whether co-transfection of PROTECTOR components can reduce off-target effects. (A) Agarose gel results of T7E1 assay for OT. The first lane contains 1kb Plus DNA Ladder (NEB) and lane 2 contains a negative control (no DNA template) reaction for the T7E1 workflow. Lanes 3, 4, and 5, contain the results from HEK293 cells that were transfected with 50 ng SpCas9 and 50 ng control scrambled gRNA plasmids, 50 ng SpCas9 and 50 ng FANCF site 2 gRNA plasmids, or 50 ng eSpCas9(1.1) and 50ng FANCF site 2 gRNA plasmids, respectively. Lanes 6-18 all received 50 ng SpCas9 and 50 ng FANCF site 2 gRNA plasmids. However, lanes 6-12 received a 10-fold excess of dSaCas9 and scrambled Sa-gRNA plasmids (500 ng each) and lanes 13-18 received a 10-fold excess of dSaCas9 and OT1-PROTECTOR gRNA (500ng each). The samples in lanes 6-9 and 13-15 were first transfected with PROTECTOR or control components and then transfected 24 h later with active CRISPR/Cas plasmids. In lanes 10-12 and 16-18, both PROTECTOR/control plasmids and active CRISPR/Cas plasmids were transfected simultaneously. The reduction of S. pyogenes (catalytically active Cas9 and gRNAs) to 50 ng was required to avoid transfection of large quantities of DNA in the 10-fold PROTECTOR excess conditions which would to widespread cell death. The samples are shown in biological triplicates. (B) Quantification of results from (A): data are presented as mean ± SEM and were quantified with ImageJ: editing efficiency is calculated as the intensity of T7E1 cleavage products divided by the sum of the intensities of T7E1 cleavage products and the uncut PCR amplicon. ** indicates $p<0.01$ by independent t-test.

Supplementary Table 1. Primers used in this manuscript.

Primer Name	Primer Sequence	Purpose
Sp_FANCF_gRNA2_mutF	gattccatgGTTTTAGAGCTAGAAATAGCAAG	Forward mutagenesis primer to synthesize pLenti-puro plasmid expressing S. pyogenes FANCF site 2 gRNA.
Sp_FANCF_gRNA2_mutR	ccttctgcagCGGTGTTTCGTCCTTTCC	Reverse mutagenesis primer to synthesize pLenti-puro plasmid expressing S. pyogenes FANCF site 2 gRNA.
Sp_CCR5_6_mutF	gtgagtagagGTTTTAGAGCTAGAAATAGCAAG	Forward mutagenesis primer to synthesize pLenti-puro plasmid expressing S. pyogenes CCR5 gRNA 6.
Sp_CCR5_6_mutR	tggtgttcatCGGTGTTTCGTCCTTTCC	Reverse mutagenesis primer to synthesize pLenti-puro plasmid expressing S. pyogenes CCR5 gRNA 6.
Sa_gBlock	TGTACAAAAAAGCAGGCTTTAAAGGAACCAATTCAGTCGACTGGATCCGGTACCAAGGTCGGGCAGGAAGAGGGCCTATTTCCCATGATTCCTTCATATTTGCATATACGATACAAGGCTGTTAGAGAGATAATTAGAATTAATTTGACTGTAAACACAAAGATATTAGTACAAAATACGTGACGTAGAAAGTAATAATTTCTTGGGTAGTTTGCAGTTTTAAAATTATGTTTTAAAATGGACTATCATATGCTTACCGTAACTTGAAAGTATTTCGATTTCTTGGCTTTATATATCTTGTGGAAAGGACGAAACACCGGAATCCCTTCTGCAGCATAGTTTTAGTACTCTGGAAACAGAATCTACTAAAACAAGGCAAAATGCCGTGTTTATCTCGTCAACTTGTTGGCGAGATTTTTTTCTAGACCCAGCTTTCTTGTACAAAGTTGGCATTA	gBlock gene fragment encoding U6 promoter, S. aureus gRNA sequence, and scaffold.
CCR2_Sa-gRNA_mutF	gagcggaggcGTTTTAGTACTCTGGAAACAG	Forward mutagenesis primer to synthesize pLenti-puro plasmid expressing S. aureus / PROTECTOR gRNA for CCR2 protection.
CCR2_Sa-gRNA_mutR	tactcgctggCGGTGTTTCGTCCTTTCC	Reverse mutagenesis primer to synthesize pLenti-puro plasmid expressing S. aureus / PROTECTOR gRNA for CCR2 protection.
FANCF_OT1_Sa-gRNA_mutF	gattccaagGTTTTAGTACTCTGGAAACAG	Forward mutagenesis primer to synthesize pLenti-puro plasmid expressing S. aureus / PROTECTOR gRNA for FANCF-OT1 protection.
FANCF_OT1_Sa-gRNA_mutR	ccttctgcagCGGTGTTTCGTCCTTTCC	Reverse mutagenesis primer to synthesize pLenti-puro plasmid expressing S. aureus / PROTECTOR gRNA for FANCF-OT1 protection.
T7R_Long_Sp	AAAAAAGCACCGACTCGGTGCCACTTTTTCAAGTTGATAACGGACTAGCCTTATTTTAACTTGCTATTTCTAGCTCTAAAAC	Universal reverse oligonucleotide, used to anneal to all gRNA-specific T7FVar oligonucleotides for S. pyogenes; used for in vitro transcription of gRNAs.
T7R_Long_Sa	AAAAAATCTCGCCAACAAGTTGACGAGATAAACACGGCATTTTGCCTTGTTTTAGTAGATTCTGTTTCCAGAGTACTAAAAC	Universal reverse oligonucleotide, used to anneal to all gRNA-specific T7FVar oligonucleotides for S. aureus; used for in vitro transcription of gRNAs.
T7F_Amp_Sp_and_Sa	GGATCCTAATACGACTCACTATAG	Universal forward amplification primer to amplify the annealed T7Fvar+T7Rlong. Works for both S. pyogenes and S. aureus. Used in combination with species-specific T7R_amp primer; used for in vitro transcription of gRNAs.
T7R_Amp_Sp	AAAAAAGCACCGACTCGG	Universal reverse amplification primer to amplify the annealed T7Fvar+T7Rlong for S. pyogenes gRNAs only; used for in vitro transcription of gRNAs.
T7R_Amp_Sa	AAAAAATCTCGCCAACAAGT	Universal reverse amplification primer to amplify the annealed T7Fvar+T7Rlong for S. aureus gRNAs only; used for in vitro transcription of gRNAs.
T7Fvar_piggyBac_Sp	GGATCCTAATACGACTCACTATAGCCCTGGGGGCTTTGGGGGGGTTTTAGAGCTAGAA	Forward oligonucleotide encoding T7 promoter and S. pyogenes gRNA targeting two sites in piggyBac plasmid, to anneal to T7R_Long_Sp; used for in vitro transcription of gRNAs.
T7Fvar_piggyBac_Sa	GGATCCTAATACGACTCACTATAGCTGTCCCTGATATCTATAACGTTTTAGTACTCTGG	Forward oligonucleotide encoding T7 promoter and S. auereus gRNA to protect the off-target site in piggyBac plasmid, to anneal to T7R_Long_Sa; used for in vitro transcription of gRNAs.
T7Fvar_scrambled_Sp	GGATCCTAATACGACTCACTATAGCACTACCAGAGCTAACTCAGTTTTAGAGCTAGAA	Forward oligonucleotide encoding T7 promoter and S. pyogenes non-targeting control gRNA, to anneal to T7R_Long_Sp; used for in vitro transcription of gRNAs.
T7Fvar_scrambled_Sa	GGATCCTAATACGACTCACTATAGCACTACCAGAGCTAACTCAGTTTTAGTACTCTGG	Forward oligonucleotide encoding T7 promoter and S. auereus non-targeting control gRNA, to anneal to T7R_Long_Sa; used for in vitro transcription of gRNAs.

Supplementary Table 2. Expected digestion products of T7E1 assays.

Primer Name	Sequence	Product size (bp)	Sp-gRNA cut site
CCR5_T7E1_F	GTTTGCATTCATGGAGGGCA	529	190
CCR5_T7E1_R	CACTTGTCACCACCCCAAAG		
CCR2_T7E1_F	GGTGTCGGAAAATGGCTGTT	573	404
CCR2_T7E1_R	TCATTTGCAGCAGAGTGAGC		
FANCF_T7E1_F	TGCTGACGTAGGTAGTGCTT	600	75
FANCF_T7E1_R	GAAGGACAATGTGAAGGCCC		
FANCF_OT1_T7E1_F	GGTGGGAGGGATGTTCTTGA	518	148
FANCF_OT1_T7E1_R	TCACAGACGCTCGCATCTAT		

Uncropped agarose gel from Figure 1C.

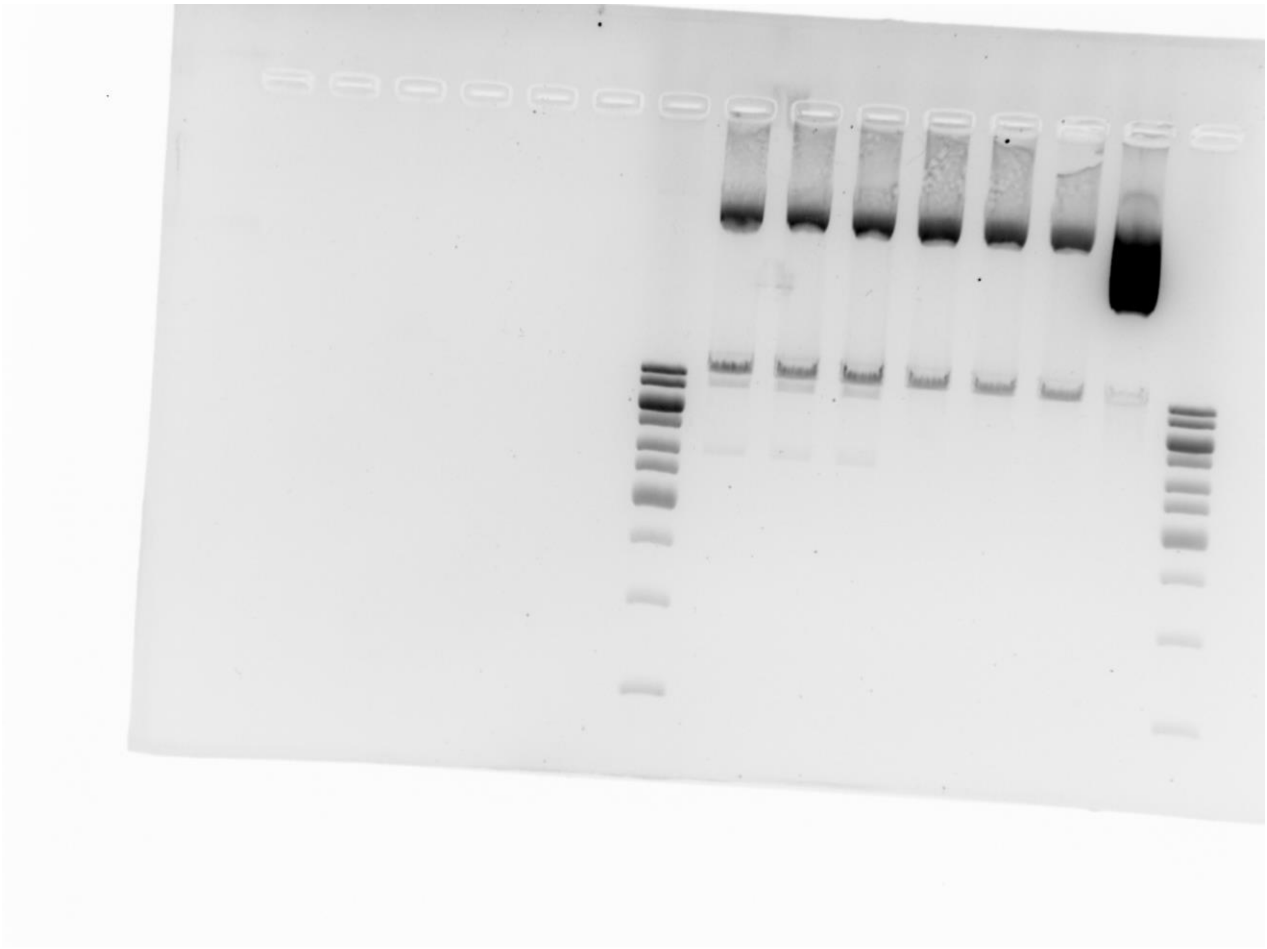

Uncropped agarose gel from Figure 2B.

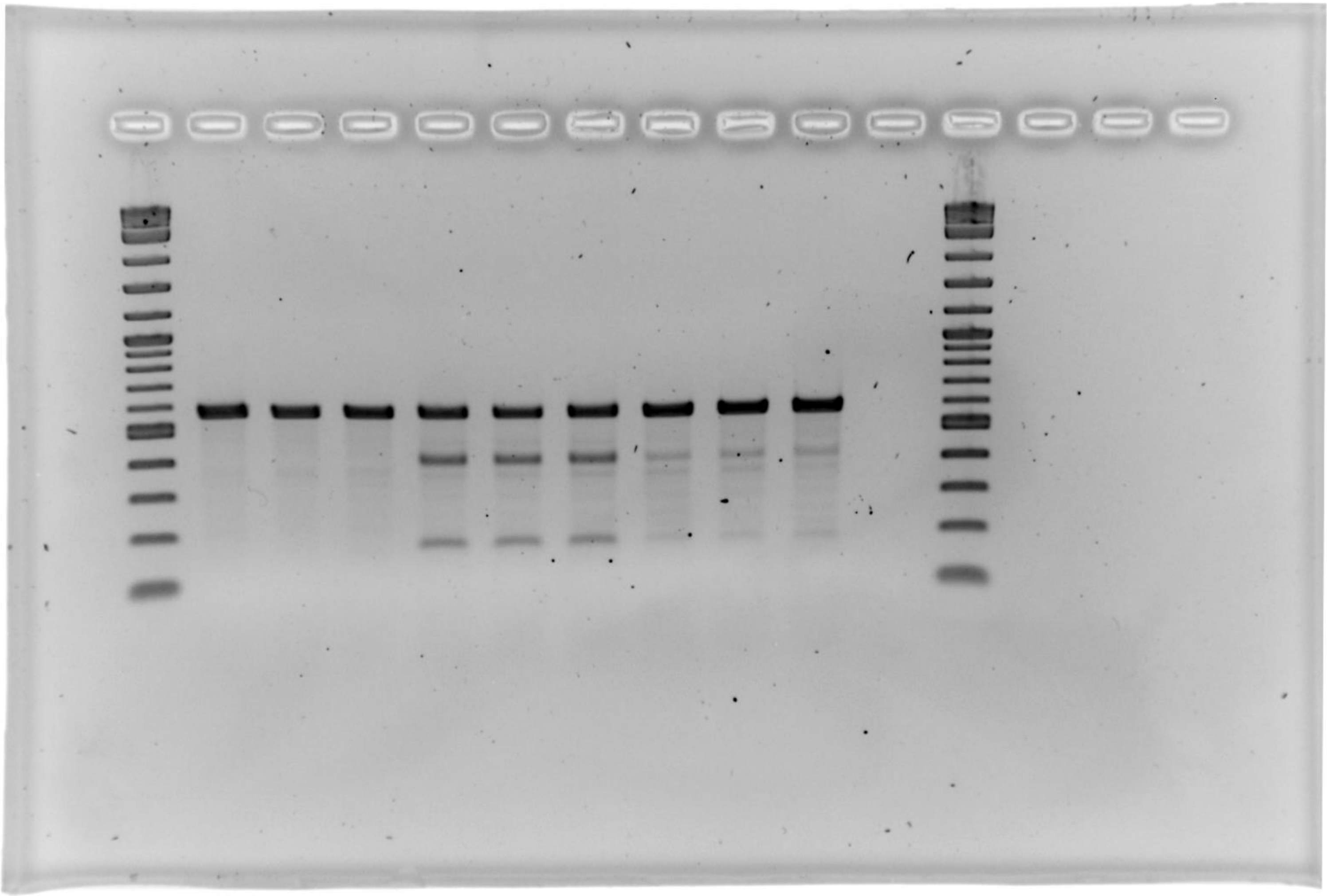

Uncropped agarose gel from Figure 2D.

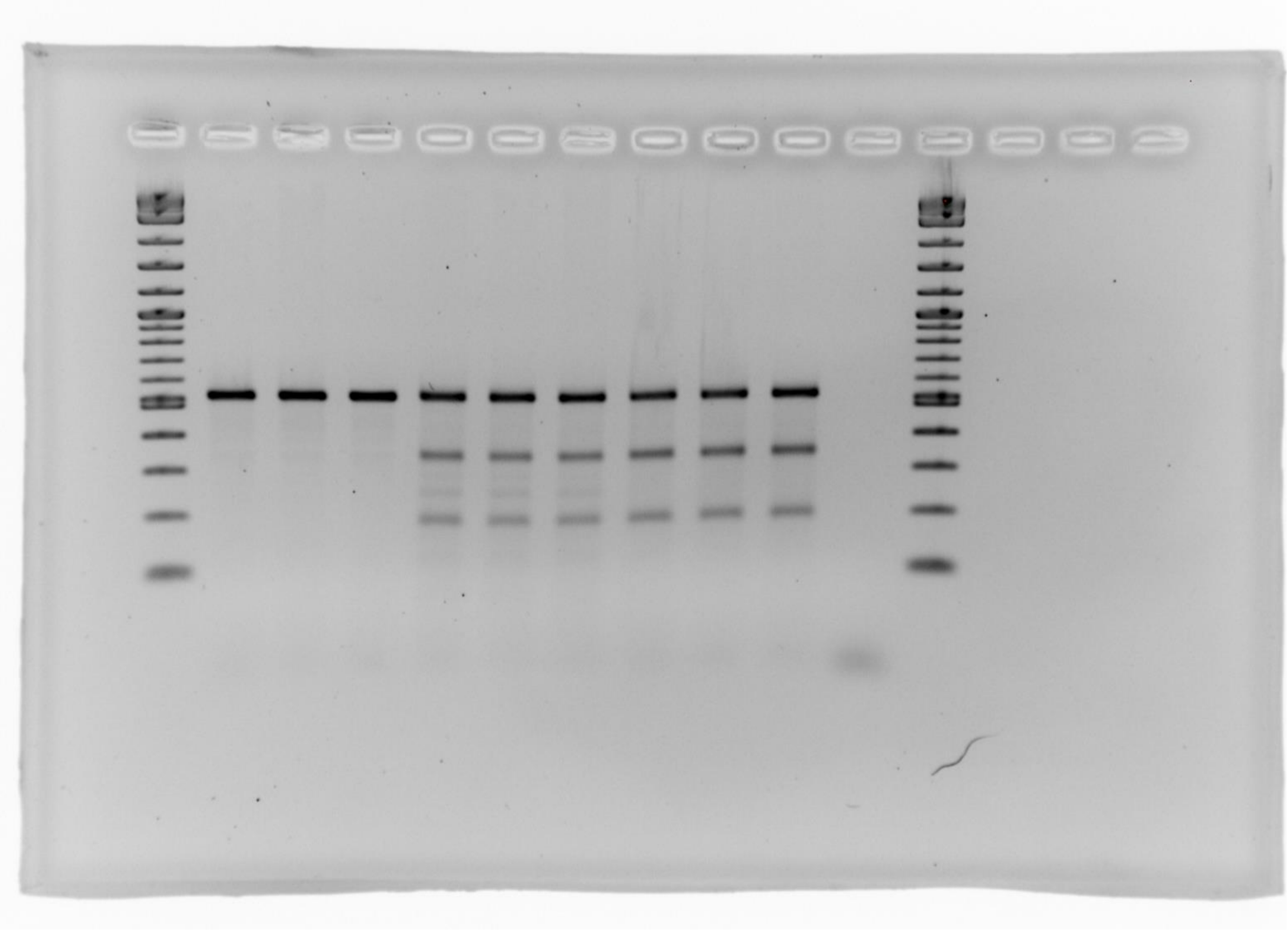

Uncropped agarose gel from Figure 3B.

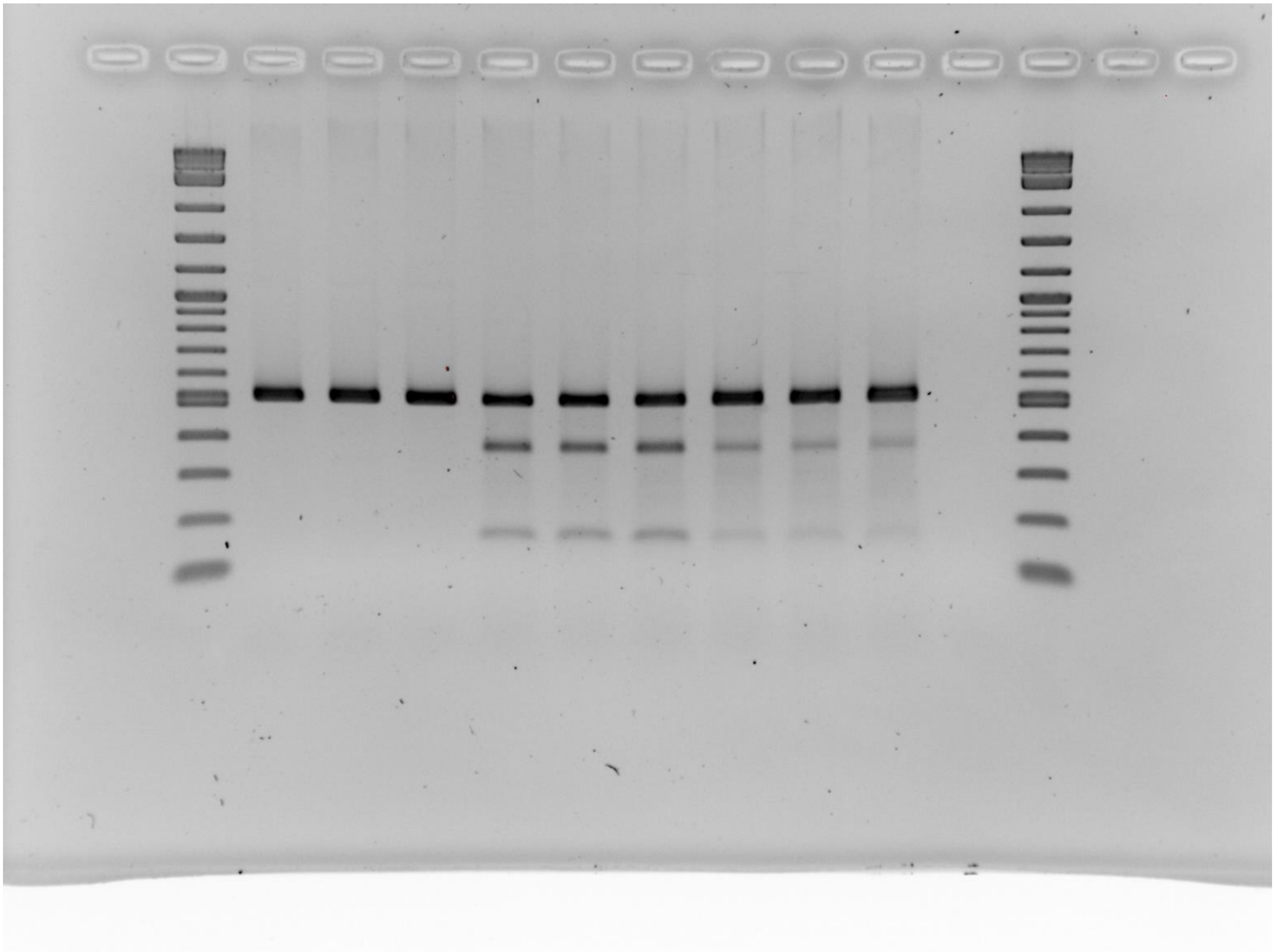

Uncropped agarose gel from Figure 3D.
Note that the additional 3 bands in the right 3 lanes (cropped out of the published figure) were undigested (no T7E1) controls used to verify that the low level of digestion by T7E1 seen in control (left 3 lanes after marker) were due to digestion by T7E1 and not mis-amplification during PCR.

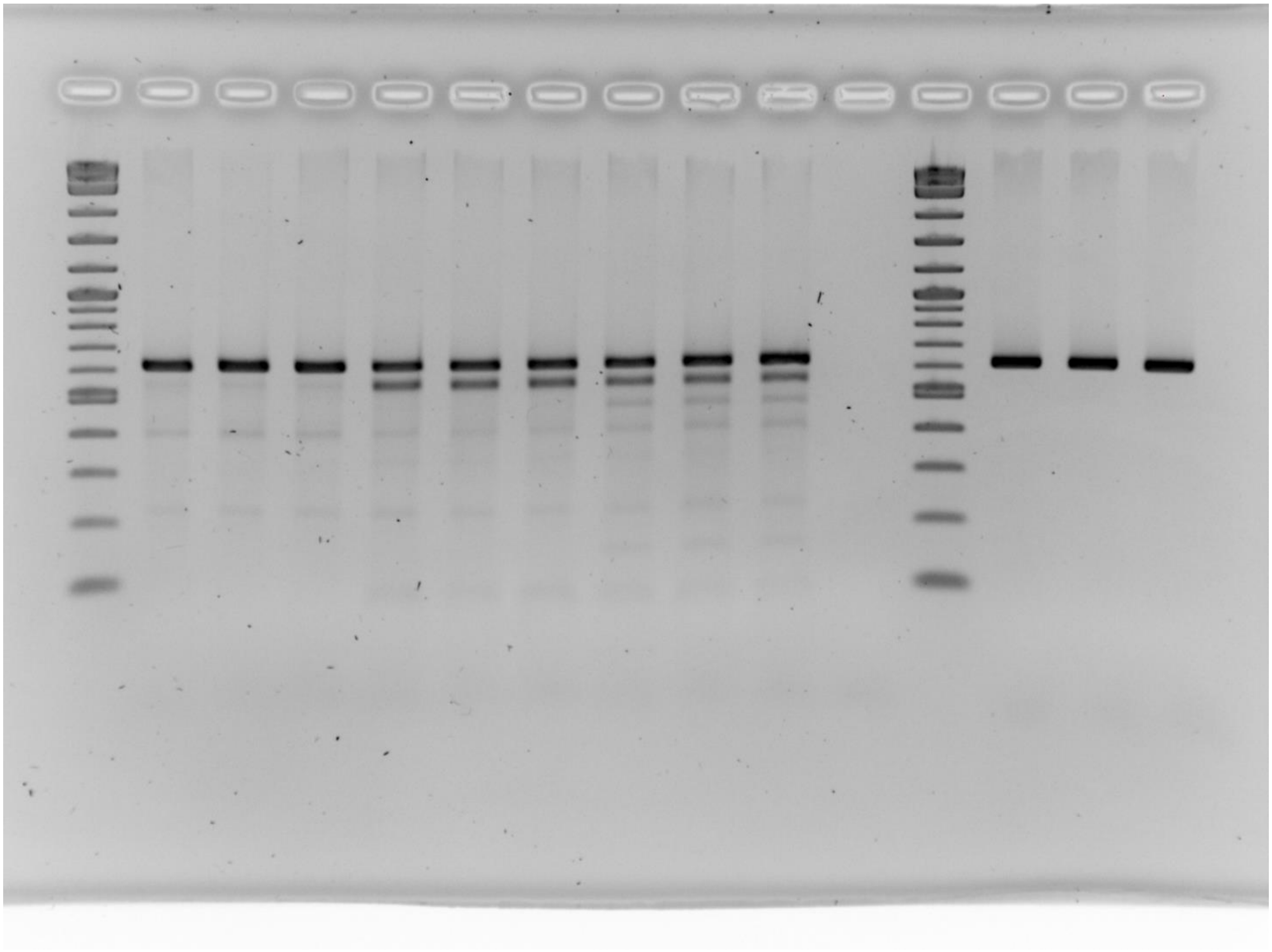

Uncropped agarose gel from Figure 3F.

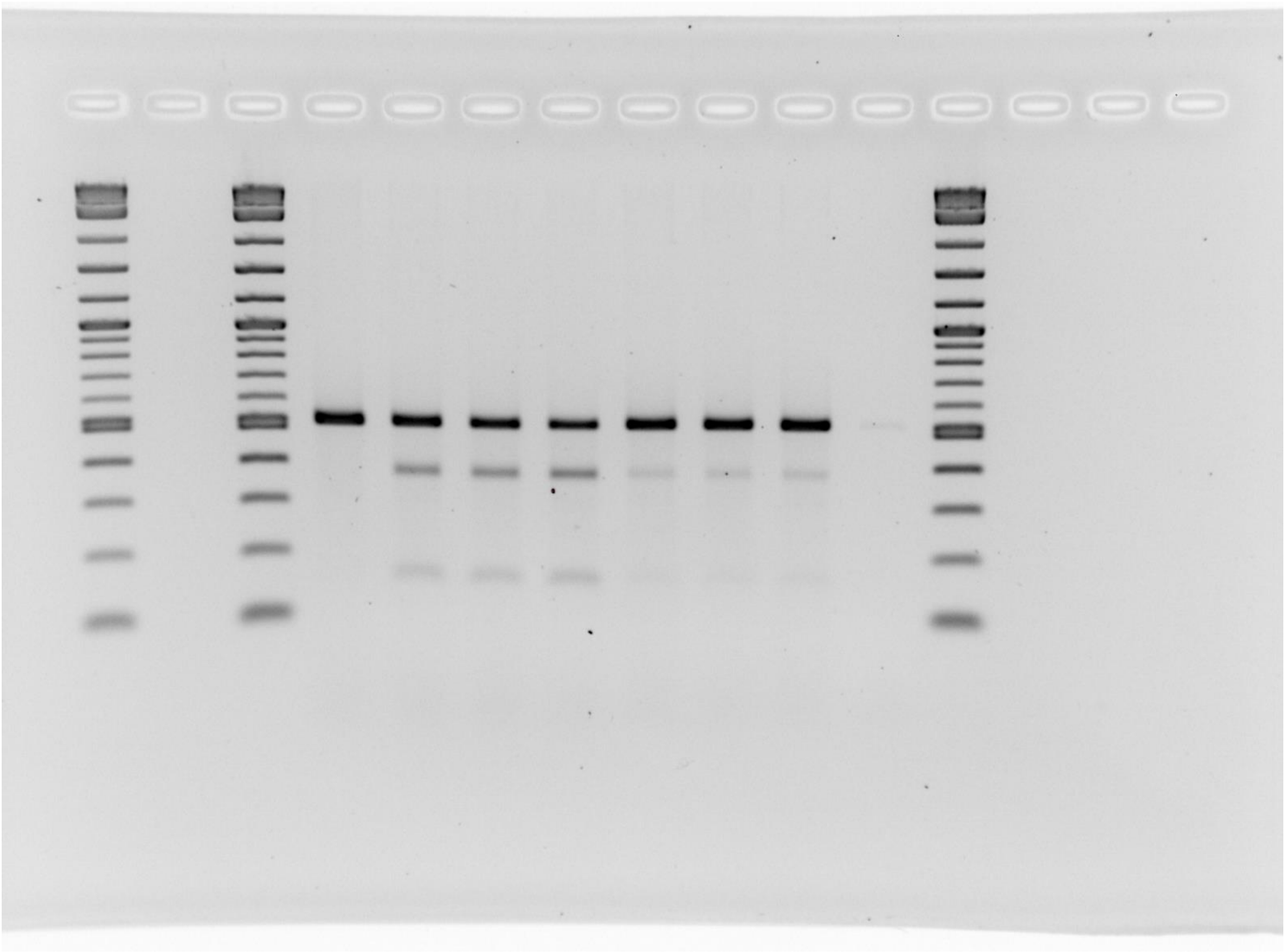

Uncropped agarose gel from Figure 3H.

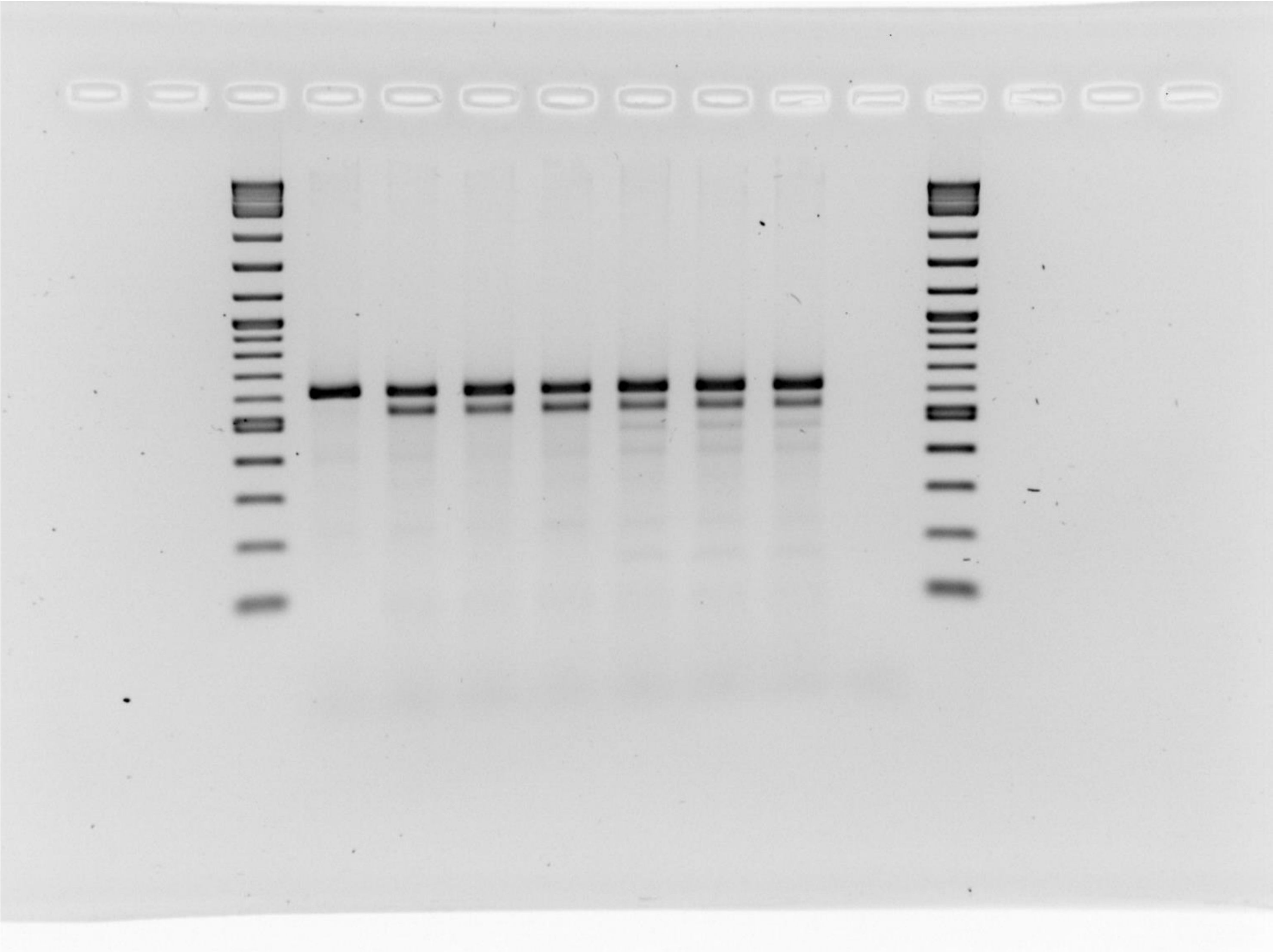

Uncropped agarose gel from Supplementary Figure 1A.

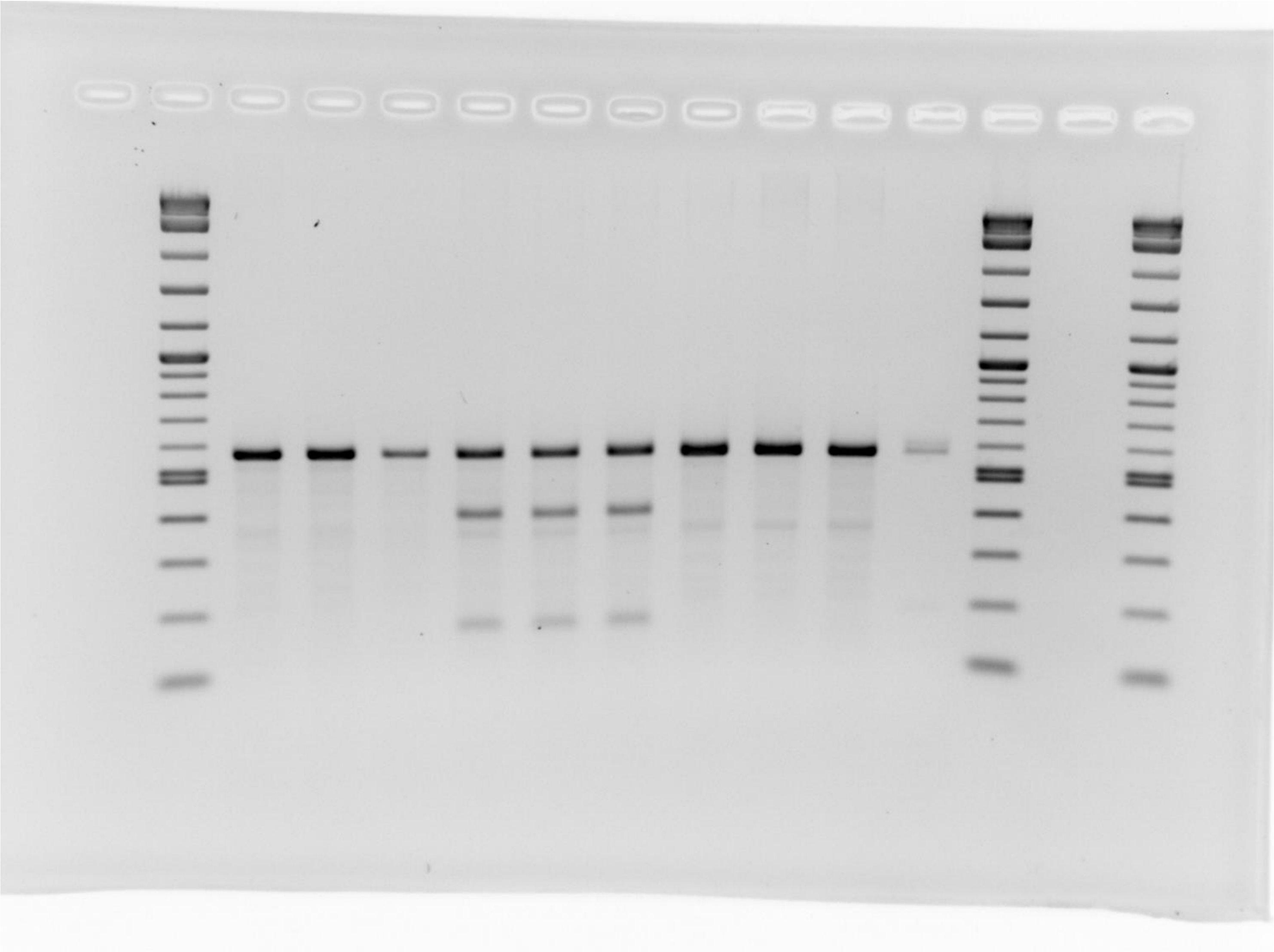

Uncropped agarose gel from Supplementary Figure 1C.

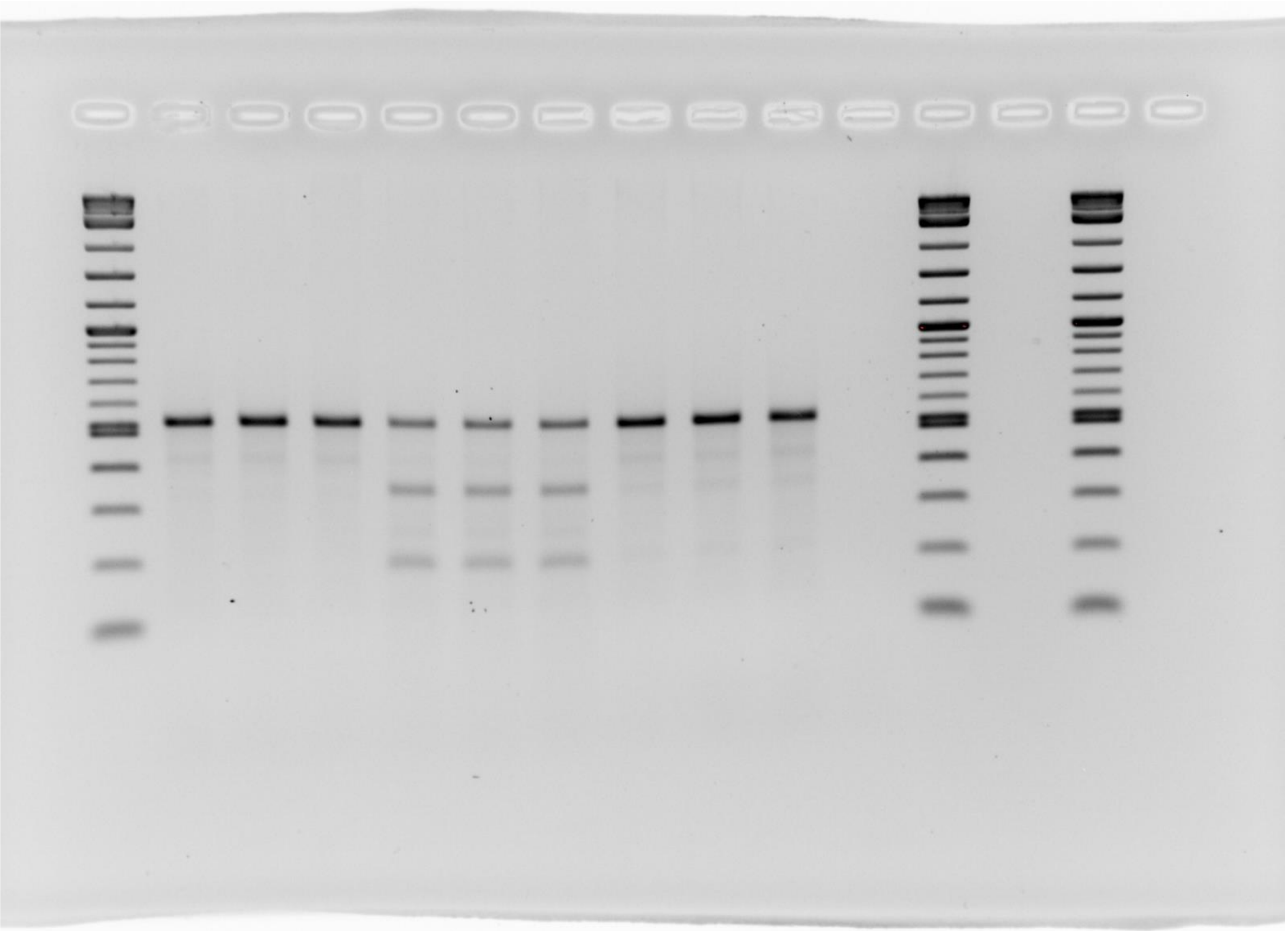

Uncropped agarose gel from Supplementary Figure 3A.

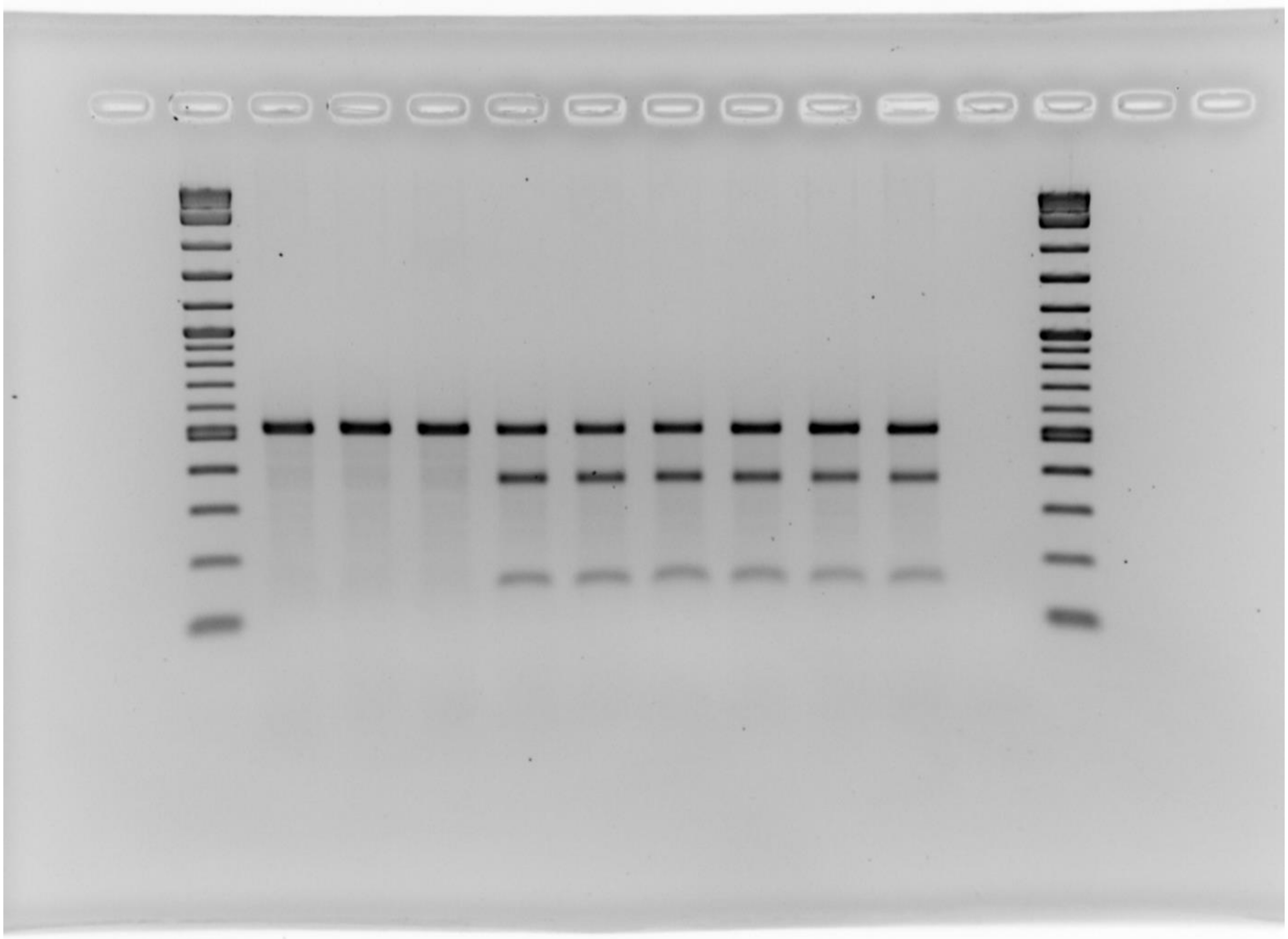

Uncropped agarose gel from Supplementary Figure 3C.

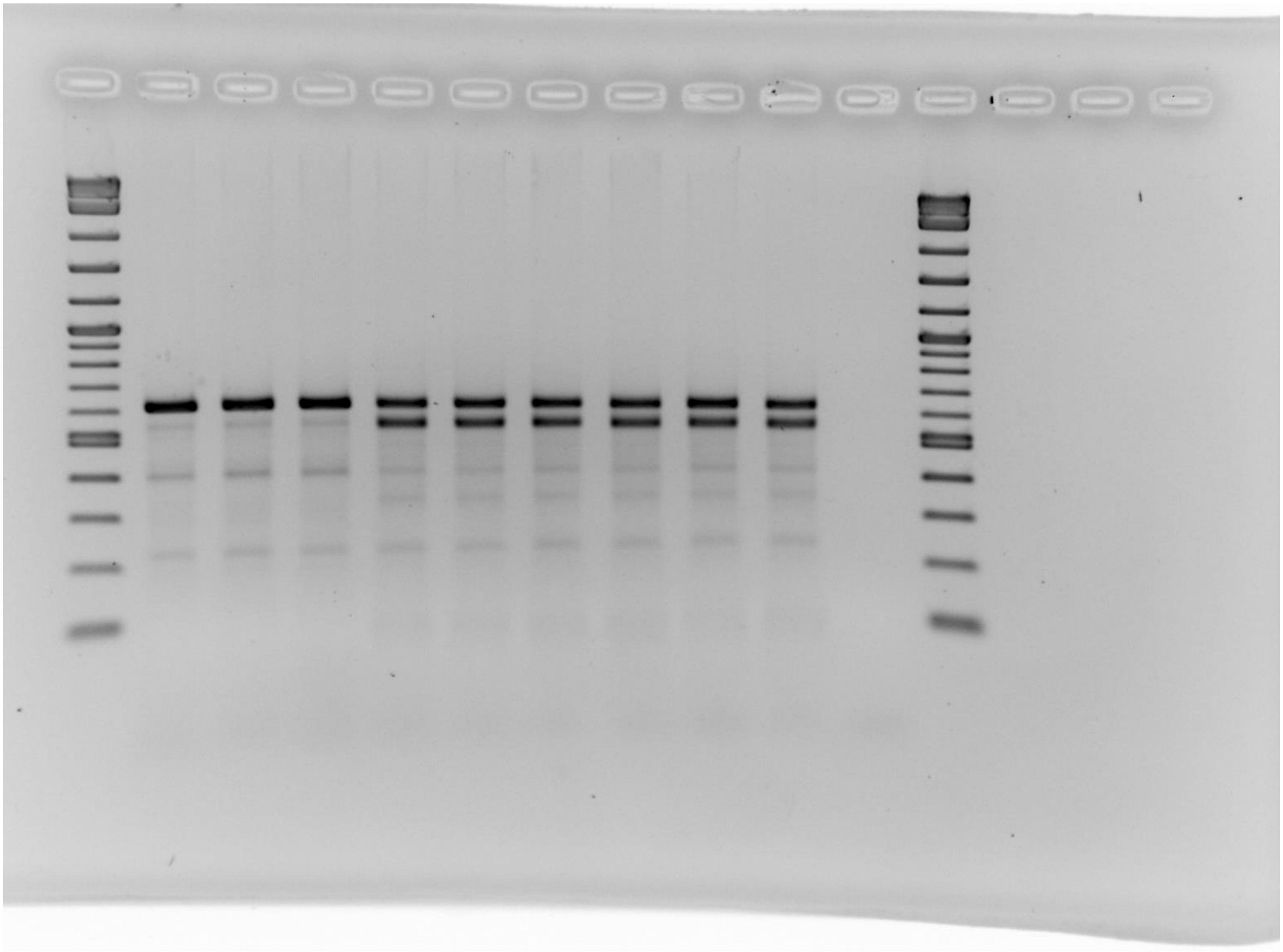

Uncropped agarose gel from Supplementary Figure 4A.

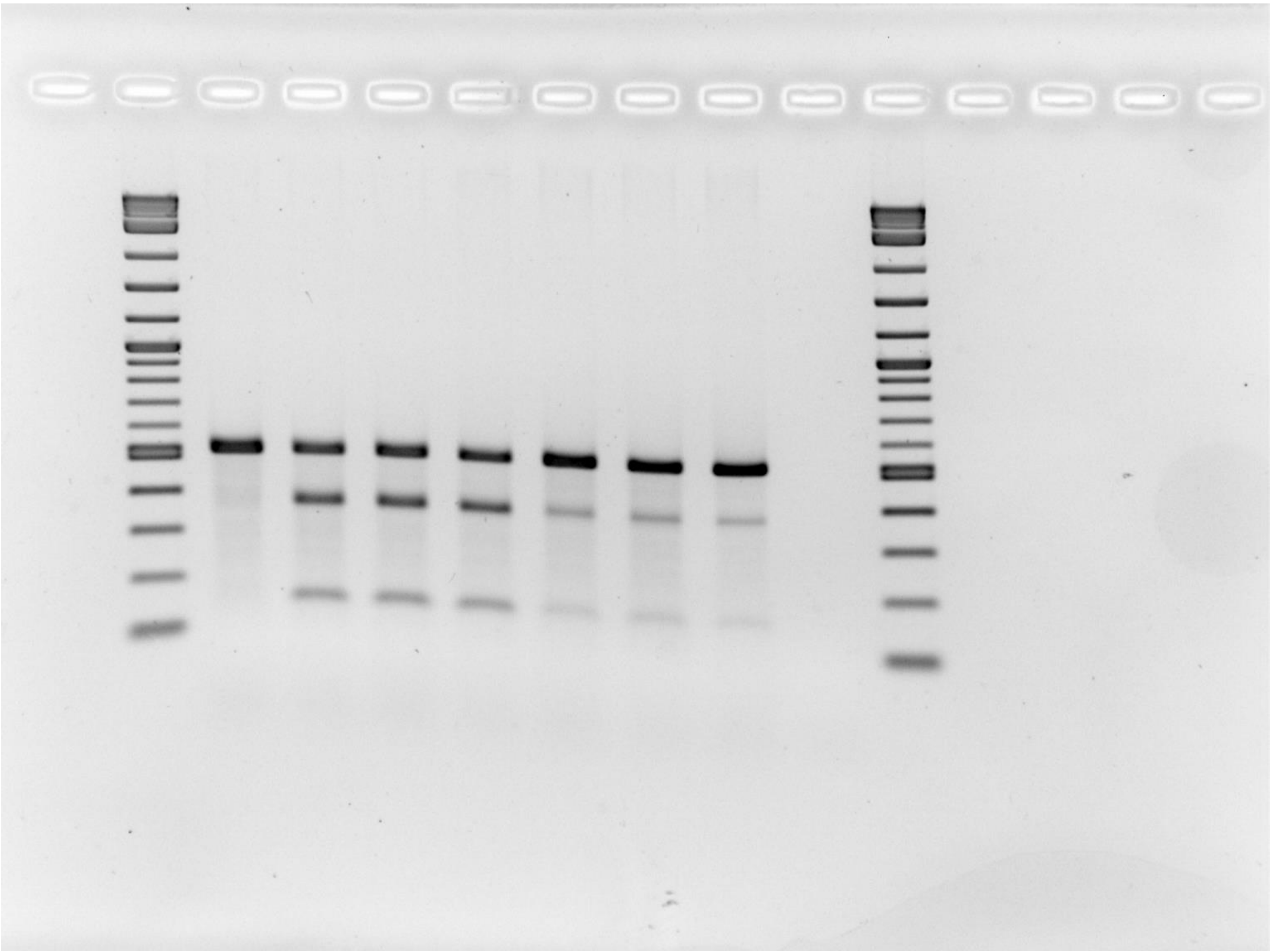

Uncropped agarose gel from Supplementary Figure 4C.

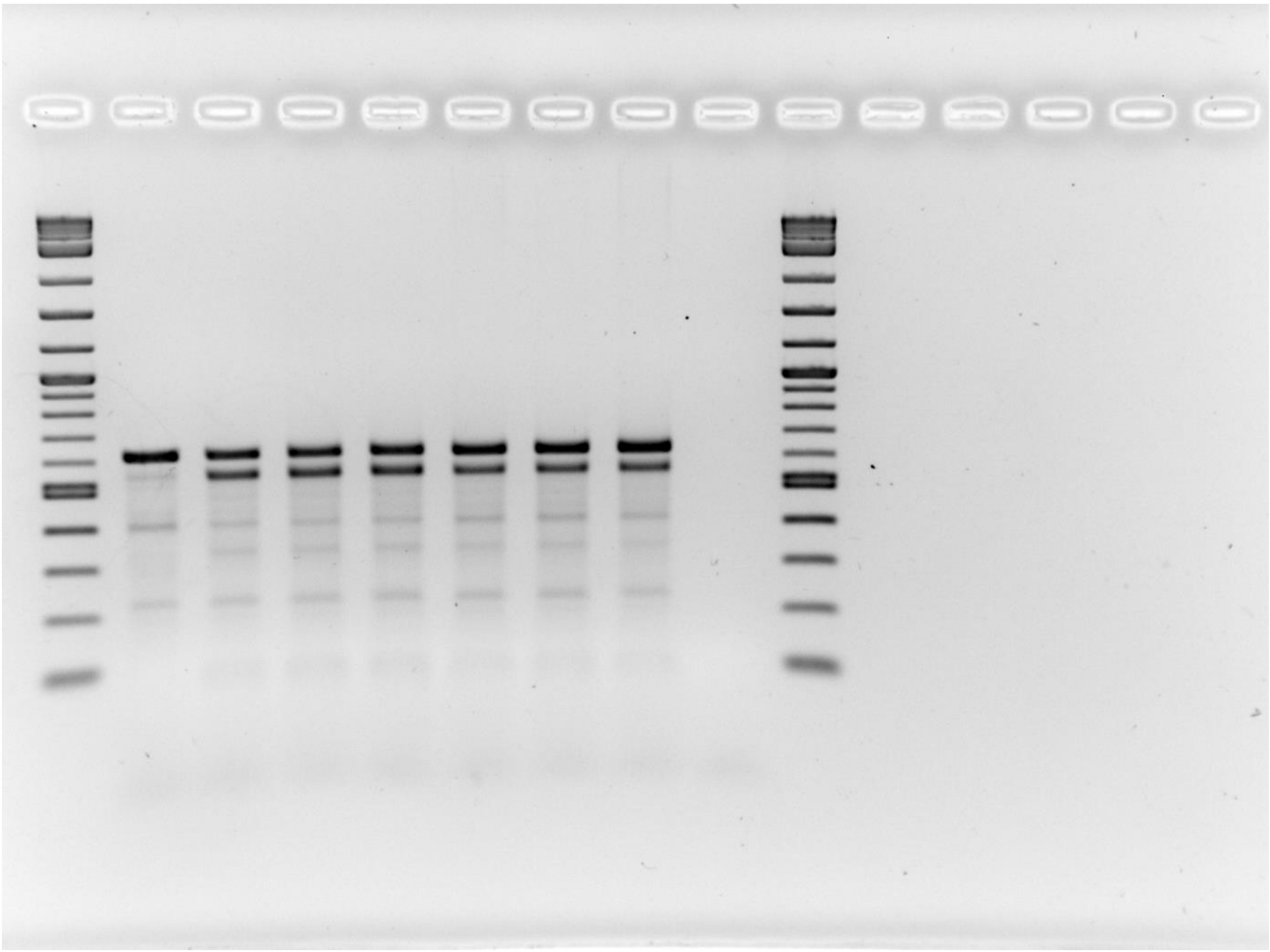

Uncropped agarose gel from Supplementary Figure 4E.

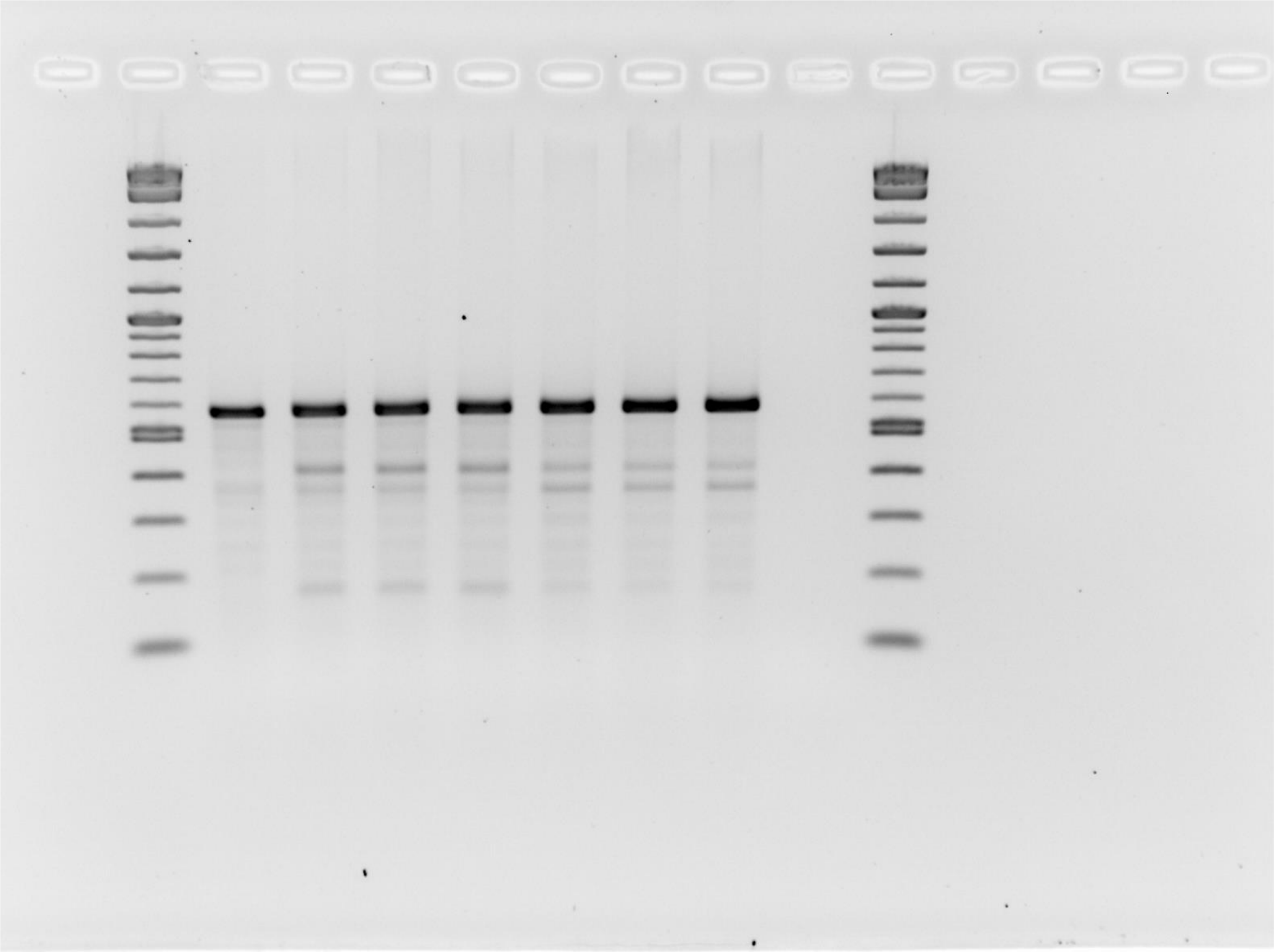

Uncropped agarose gel from Supplementary Figure 4G.

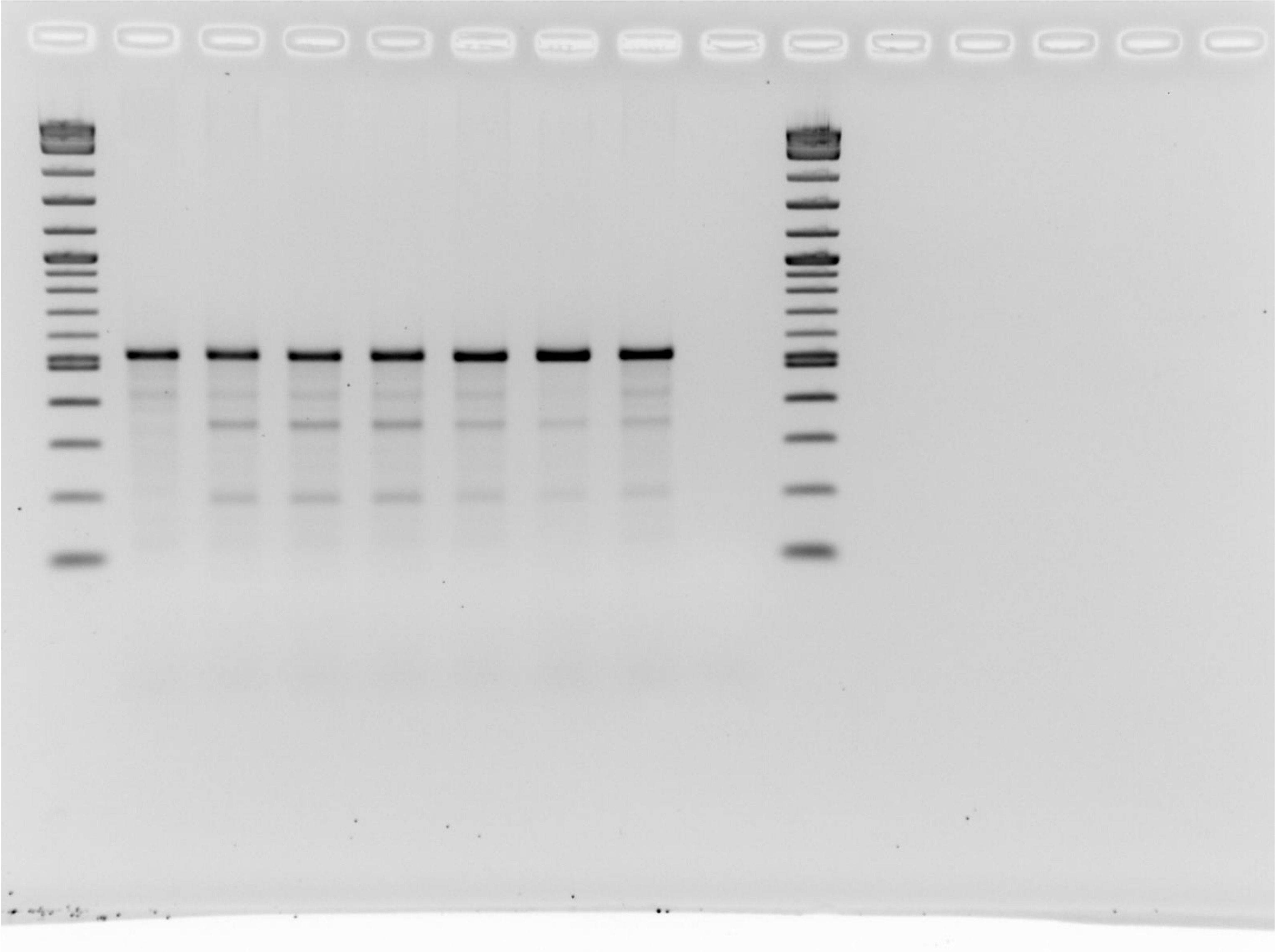

Uncropped agarose gel from Supplementary Figure 5A.

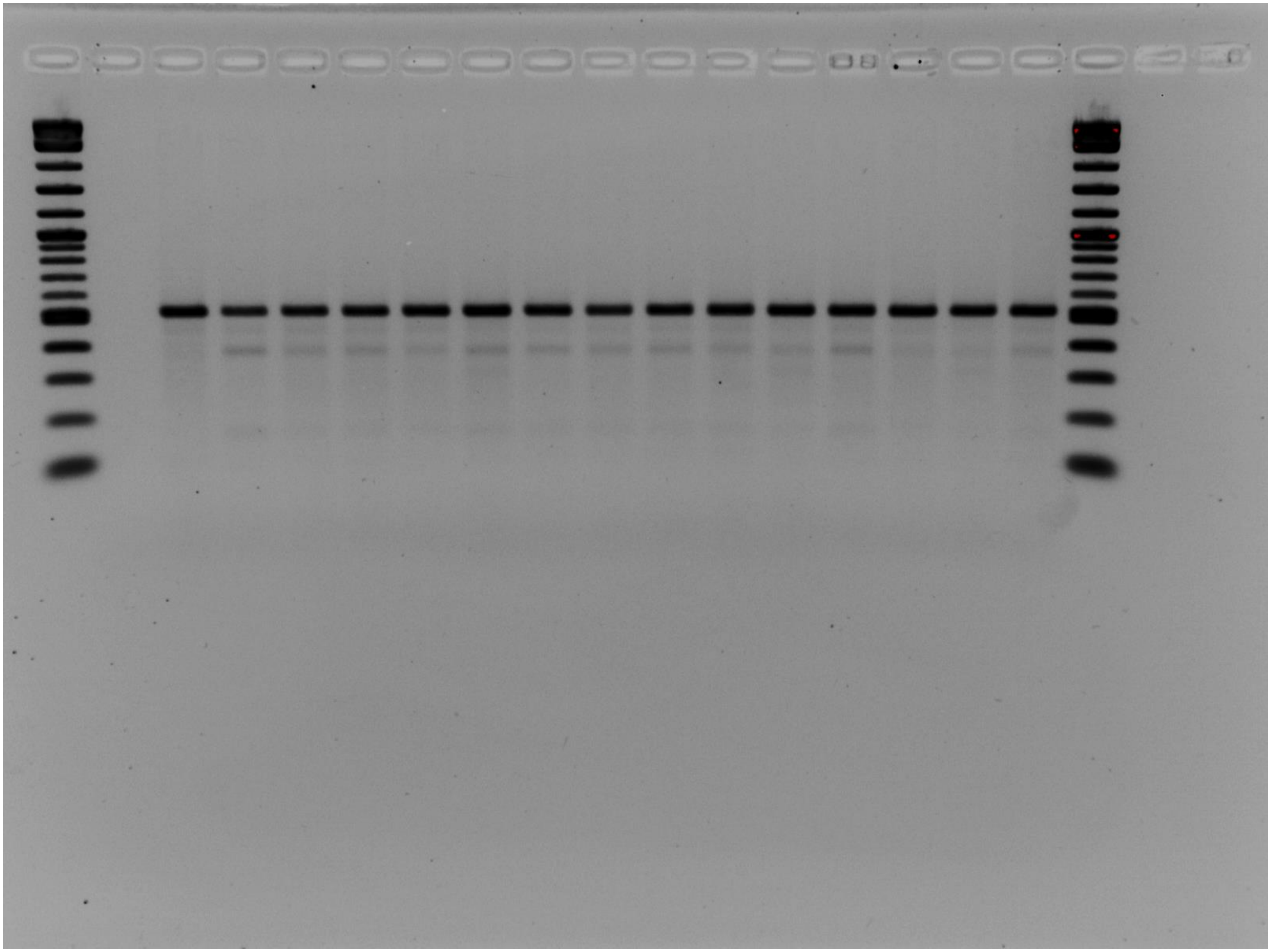

Supplement: Supplementary file 1 — Supplementary Information. [file 41598_2023_29332_MOESM1_ESM.pdf]
